# Beyond Isolated Optimization: A Holistic Review Across the Pre-Mid Post-Treatment Chain for Hard Carbon in Sodium-Ion Battery

**DOI:** 10.1007/s40820-026-02248-y

**Published:** 2026-06-05

**Authors:** Qingxuan Geng, Yonghui Zhang, Dongxu Xie, Chenhui Hao, Liping Guo, Jiwei Zhang, Paul K. Chu, Qingwei Li

**Affiliations:** 1 State Key Laboratory of Green Papermaking and Resource Recycling, Advanced Research Institute for Multidisciplinary Science, Qilu University of Technology (Shandong Academy of Sciences), Jinan, 250353 People’s Republic of China; 2 National & Local Joint Engineering Research Center for Applied Technology of Hybrid Nanomaterials, Henan University, Kaifeng, 475004 People’s Republic of China; 3 Department of Physics, Department of Materials Science and Engineering, and Department of Biomedical Engineering, City University of Hong Kong, Tat Chee Avenue, Kowloon, Hong Kong, People’s Republic of China; 4 Institute of Advanced Energy Materials and Technology, School of Metallurgy and Energy, Wuhan University of Science and Technology, Wuhan, 430081 People’s Republic of China

**Keywords:** Hard carbon, Synergistic optimization, Microstructural regulation, Sodium-ion battery, Energy storage

## Abstract

Commercialization of sodium-ion batteries depends on the advancement of high-performance hard carbon (HC) anodes. Their complex microstructure, composed of graphitic nanodomains, nanopores, and defects, fundamentally governs sodium storage. However, most design of HC is hampered by a fragmented research approach focusing on single-point optimization. Isolated improvements in the precursor selection, pyrolysis, or post-treatment often produce unsatisfactory results due to overlooked synergistic effects across the entire fabrication chain. This review addresses this challenge by proposing a holistic, full-process engineering perspective. We first clarify how the core structural features of HC collectively determine the storage mechanism. We then systematically examine the roles of pretreatment, mid-process control, and post-treatment in microstructural manipulation. We also critically assess and comparatively evaluate these strategies by identifying their respective strengths, shortcomings, and interdependencies. Our analysis emphasizes that treatments at one stage precondition outcomes at another, highlighting key coupling points for synergistic optimization. How to coordinate these strategies in industrial production to obtain batch performance stable HC has also been discussed. Finally, we put forward future research directions, focusing on establishing quantitative microstructure–property relationships through advanced characterization and modeling, developing cross-stage co-design strategies, and evaluating process scalability and sustainability. This review aims to guide the transition of HC anode development from exploratory confusion toward rational, predictive engineering.

## Highlights

- Proposes a holistic “Pre-Mid-Post” full-process engineering mode to go beyond fragmented single-point optimization of hard carbon anodes
- Elucidates the synergistic and contradictory interplay among graphitic domains, nanopores, and defects in determining the Na⁺ storage properties
- Future design necessitates cross-stage co-optimization and quantitative microstructure–performance relationships for rational HC engineering

## Graphical abstract

**Figure:**
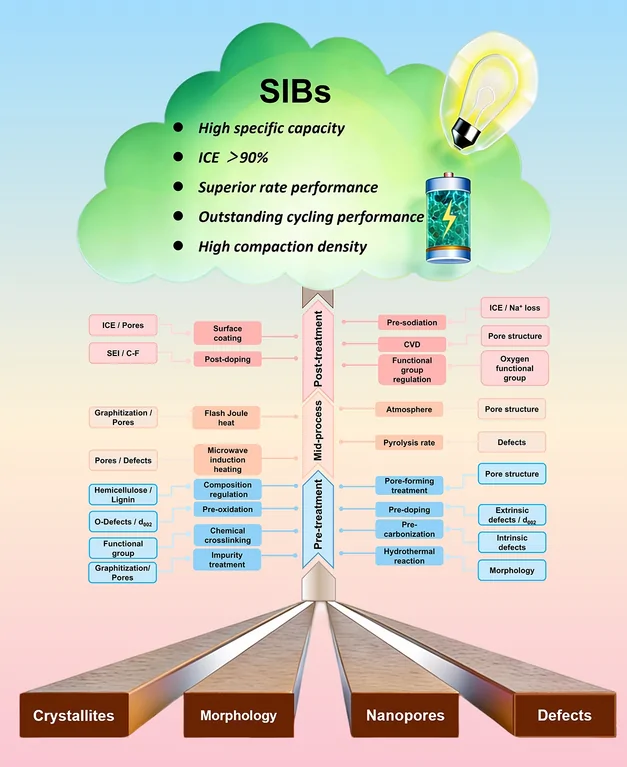

## Introduction

Lithium-ion batteries heavily rely on lithium, cobalt, and nickel resources, which are relatively scarce [1–3]. Notably, lithium is far less abundant than sodium, and its uneven geographical distribution leads to significant price volatility influenced by policy. Consequently, low-cost sodium-ion batteries (SIBs) have been developed. Technically, SIBs offer advantages over lithium-ion batteries, including better low-temperature performance, enhanced safety, and faster charging capabilities, making them more suitable for large-scale energy storage in cold climates [4–6]. Given the urgent global need for energy transition and expanded energy storage capacity, SIBs present significantly improved economic viability and sustainability compared to their lithium-ion counterparts [7, 8]. The key to transitioning SIBs from laboratory research to large-scale commercial application lies in the design, preparation, and technological maturity of their electrode materials.

The design and preparation of anode materials are crucial to the energy density, cycle life, and overall cost of SIBs [9, 10]. Unlike the graphite anode used in lithium-ion batteries, the larger ionic radius of sodium ions prevents their effective intercalation into the ordered interlayer spaces of graphite [11]. This results in very low capacities, rendering traditional graphite anodes nearly ineffective for sodium storage [12]. Therefore, developing anode materials capable of storing sodium ions efficiently, stably, and reversibly is crucial [13–17]. In recent years, various anode materials, such as alloys, metal oxides/sulfides, and HC (HC), have been explored [18]. Among these, HC has emerged as the most promising and, to date, the only commercially viable anode material for SIBs [19].

HC is typically a non-graphitizable carbon produced by pyrolyzing organic precursors at moderate temperatures (usually between 1000 and 1500 °C) [20]. It possesses a unique “house-of-cards” microstructure characterized by randomly oriented, curved graphitic-like domains, expanded interlayer spacing, and a high concentration of nanopores and defects [21]. This disordered structure provides ideal storage sites and diffusion pathways for the larger Na⁺ ions, enabling reversible sodium storage. HC offers a balanced combination of advantages in terms of specific capacity, working potential, structural stability (with minimal volume change), abundance of low-cost precursors, and scalable manufacturing processes [22]. Consequently, it has become the anode material of choice for commercial SIBs. Despite its promise, HC still faces several key scientific and technical obstacles. Its large specific surface area, defects, and surface oxygen-containing functional groups can lead to the formation of a thick and unstable solid electrolyte interphase (SEI) during the initial discharge [23–25]. This process consumes significant amounts of electrolyte and sodium ions, causing a low initial Coulombic efficiency (ICE) [26]. Furthermore, its inherently low electronic conductivity and disordered structure hinder fast electron transport, while suboptimal pore structures and tortuous ion diffusion paths result in sluggish sodium-ion kinetics and poor rate performance [27]. Inconsistencies in precursor materials directly lead to batch-to-batch variations and performance fluctuations in the final HC product. Additionally, sodium metal may deposit unevenly at very low potentials, leading to dendrite formation and dead sodium, which accelerates capacity fading. Other issues include a moderately low specific capacity and, particularly, a low tap density, which limits volumetric energy density [28].

To address these challenges, extensive research has been conducted. Strategies include precursor composition control, pre-oxidation/pre-carbonization, doping, optimization of pyrolysis conditions, post-synthesis coating, composites with soft carbon, and investigations into storage mechanisms [29–31]. These areas represent the forefront of current HC research and are crucial to its advancement. For instance, Wang et al. have shown that highly crystalline cellulose in natural wood precursors forms long-range carbon layers acting as closed-pore walls during carbonization, while amorphous components inhibit graphitization and cause these layers to become brittle [32]. Huang et al. have proposed a kinetically decoupled carbonization strategy combining low-temperature pyrolysis with rapid high-temperature Joule heating [33]. This method allows precise control over impurity removal, promotes grain growth, and prevents interlayer spacing contraction, yielding high-performance HC. Xie et al. have employed low-pressure chemical vapor deposition (CVD) to perform in situ growth of a modified layer composed of epitaxially grown crumpled graphene on the HC surface [34]. The surface crumpled graphene endows HC with enhanced electronic and ionic conductivity. It also effectively passivates the intrinsic surface defects of the substrate, thereby facilitating a notable boost in ICE. While single-factor optimization can undoubtedly improve certain properties, HC properties are influenced by a multitude of interconnected factors. These include defects, chemical environment, pores (open/closed, distribution, size, structure), pseudo-graphitic domains (length, interlayer spacing, number of layers, quantity), amorphous regions, graphitic-like domains, and morphology [35, 36]. Therefore, it is difficult to construct an ideal HC material by controlling just one parameter. A systematic and holistic understanding of the entire preparation chain, from pretreatment and intermediate pyrolysis process control to post-treatment, is needed. Such a perspective is vital for guiding the rational design of next-generation HC anodes.

**Fig. 1:**
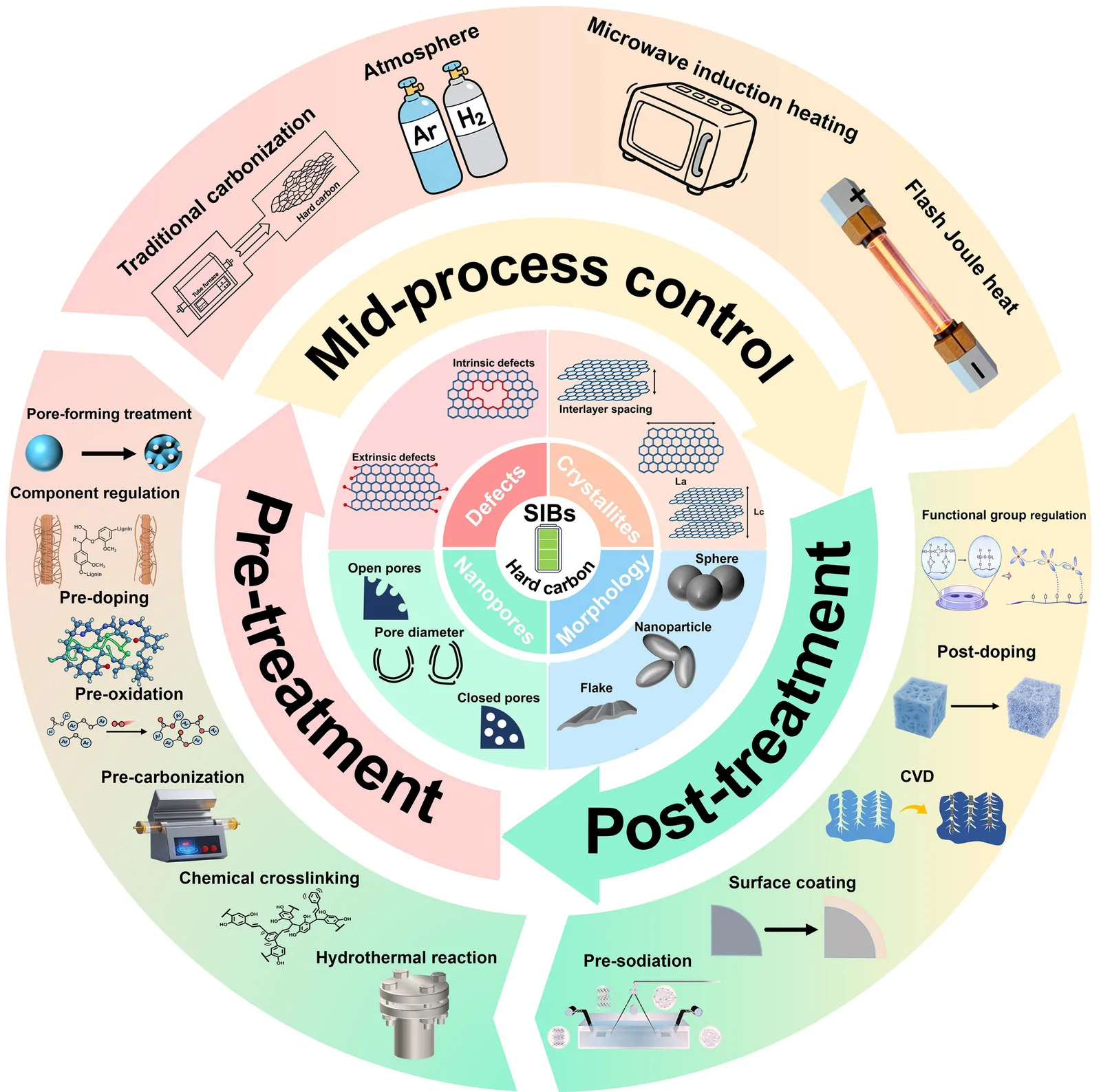
Schematic diagram of the regulation strategies for HC

This review aims to examine the complete developmental chain of HC anode preparation technology. As shown in Fig. 1, we systematically divide the design and preparation process of HC into three parts, including pretreatment engineering, mid-carbonization/pyrolysis, and post-treatment modification. The review explores how each part determines the microstructure of HC and, consequently, its electrochemical performance. We first summarize the model structures of HC and their impact on energy storage, clarifying the key influencing factors. Then from three perspectives, we analyze how fine-tuning the microstructure is achieved: (1) Pretreatment engineering, including component regulation, pre-oxidation, hydrothermal treatment, precursor doping, etc., (2) Mid-pyrolysis Processes, covering traditional slow pyrolysis, novel rapid carbonization methods, atmosphere, and temperature., (3) Post-treatment Strategies, such as surface coating, chemical modification, pore filling, and pre-sodiation. For each stage, we elucidate how the resulting microstructural features determine key performance indicators like capacity, ICE, and cycle life. Finally, we provide an outlook on the future design and development of HC. In this part, we stress the necessity of an integrated design concept that focuses on the synergistic effects across the entire preparation chain rather than isolated variables. We also highlight the importance of detailed disclosure of the physicochemical information of precursors. Furthermore, leveraging machine learning can accelerate the research and development of high-performance HC. It is also critical to consider the industrial feasibility of process combinations during R&D and to establish a multi-dimensional “dictionary” for HC design and preparation. This review aims to provide strategic guidance for researchers and engineers dedicated to advancing HC anode technology, thereby accelerating the development of commercially competitive SIBs.

## Fundamentals of HC Structure and Sodium Storage Mechanism

### Fundamentals of HC Structure

**Fig. 2:**
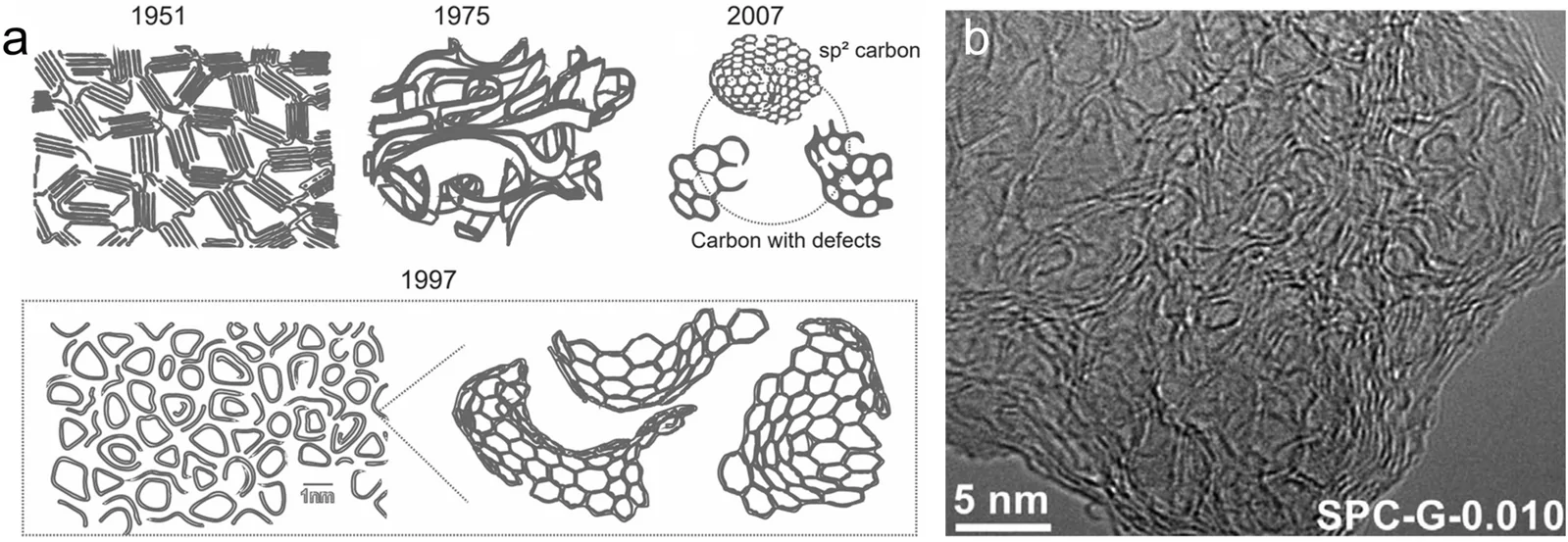
**a** Schematic diagram of the structure of HC and **b** high-resolution transmission electron microscopy image [6, Copyright 2023, The Royal Society of Chemistry. [7, Copyright 2025, Wiley]

As an anode material for SIBs, HC governs sodium storage performance via its microstructural characteristics. As shown in Fig. 2, the core characteristic of the microstructure of HC is highly disordered and diverse coexistence [6, 7]. It is not a long-range ordered graphite crystal, but is composed of bent and twisted graphene-like carbon sheets (mainly *sp*^*2*^ hybridization) stacked in a short-range ordered and long-range disordered manner, forming a “house of cards” structure [21]. The curved graphene nanosheets undergo disordered stacking in three-dimensional space via an amorphous carbon matrix [37]. This unique topological configuration endows the material with a highly disordered nanoporous network while retaining moderate graphitization features, thereby constructing multiscale sodium ion transport channels. Therefore, the microcrystals, nanopores, and defects of HC collectively determine its sodium storage properties.

#### Graphitic Microcrystals

Graphitic microcrystals constitute the core of HC, and the essence of regulating the microstructure of HC is to control the size, stacking mode, and spatial relationship between carbon microcrystals [38, 39]. Utilizing structural characterization techniques such as XRD and small-angle X-ray SAXS, researchers have established a quantitative correlation model between microcrystal parameters and sodium storage performance [40]. The dimensions of graphitic microcrystals in HC typically span from several to tens of nanometers, with this disordered arrangement not only providing interlayer galleries for sodium ion insertion but also increasing accessible sodium adsorption sites [12, 41].

A moderate increase in La within the 2–5 nm range facilitates the formation of a continuous *sp*^*2*^-hybridized conductive network, enhancing intrinsic electronic conductivity, mitigating ohmic polarization during galvanostatic charge–discharge cycling, and optimizing in-plane sodium ion diffusion pathways [42]. This *La* range also preserves abundant edge sites that act as reversible sodium adsorption centers in the high-potential sloping region, boosting overall reversible capacity. Excessively small *La* values disrupt conductive network continuity, impairing rate performance at high current densities, while *La* values exceeding 10 nm drive interlayer spacing contraction and reduce accessible edge site density, leading to a marked decline in reversible capacity [43]. Beyond interlayer spacing, the basal plane coherence length (*La*, defining lateral domain size) and c-axis stacking thickness (*Lc*, corresponding to graphene layer number) are the dominant structural parameters governing electrochemical output [41]. For the stacking dimension, a moderate *Lc* of 1.5–3 nm, corresponding to 4–8 stacked graphene layers, constructs a robust carbon framework that accommodates volumetric fluctuation during repeated sodium (de)intercalation, underpinning long-term cycling stability. Insufficient *Lc* (fewer than 3 layers) renders the carbon matrix susceptible to irreversible structural collapse during cycling, while excessive *Lc* (> 5 nm) elevates sodium ion diffusion steric hindrance and contracts interlayer spacing, slowing reaction kinetics and diminishing rate capability [44].

In addition to *La* and *Lc*, the interlayer spacing is an important factor affecting the kinetics of sodium ion transport [45]. The inherent curvature of the graphitic nanosheets attenuates interlayer van der Waals attractions, inducing expansion of the (002) interlayer spacing to beyond 0.37 nm, a value markedly exceeding the 0.335 nm spacing of pristine graphite. Cao et al. demonstrate that a 0.37 nm interlayer spacing corresponds to a sodium insertion energy barrier of 0.053 eV, sufficiently low to enable facile reversible ion intercalation [44]. Sun et al. further defined the thermodynamically feasible interlayer spacing range for efficient sodium storage as 0.36–0.4 nm, with subsequent studies confirming that expanded interlayer spacing accelerates sodium ion diffusion and enhances anode rate capability [45].

#### Nanopores

As another pivotal microstructural component of HC, nanopores are typically generated between curved graphene nanosheets, with their size and spatial distribution exerting a prominent impact on the sodium storage performance of HC [45–56]. The most critical structure–property correlation for nanoporous architectures lies in the open-to-closed pore ratio within the carbon matrix, as these two pore categories exert fundamentally distinct and even opposing effects on the electrochemical performance of HC anodes.

Closed pores, defined as nanoscale cavities isolated from the external particle surface, are the dominant structural contributors to the low-voltage plateau capacity of HC. These confined cavities enable quasi-metallic sodium filling without direct contact with the liquid electrolyte, maximizing the utilization of sodium storage sites while avoiding parasitic electrolyte decomposition within the pore interior. Beyond plateau capacity regulation, ultramicropores with dimensions below 0.7 nm enhance ICE by acting as a molecular sieve for desolvated sodium ions. Yang et al. demonstrated that the solvation structure of sodium ions within HC hosts is strongly dictated by the pore mouth size (PMS) [57]. For PMS values ranging from 0.35 to 0.5 nm, a range permissive to CO₂ adsorption while impermeable to N₂, only a fraction of coordinated solvent molecules is sieved out prior to sodium ion entry into the pore channels, with contact ion pairs identified as the predominant solvation configuration within the pore interior. In contrast, a PMS below 0.35 nm enables effective sieving of the majority of coordinated solvent molecules, giving rise to a solvation structure dominated by anion aggregates (AGGs) within the pore cavities. The near-complete exclusion of solvent molecules from the pore interior, with the solvation environment governed predominantly by AGGs, facilitates the formation of a thin, inorganic NaF-rich SEI.

Alptekin et al. reported that rational modulation of the volume and size of closed nanopores can elevate the plateau capacity to 75% of the total reversible capacity of HC [52]. In addition, mesoporous structures with dimensions ranging from 2 to 50 nm construct three-dimensionally interconnected ion transport networks, which optimize electrolyte wettability, reduce sodium ion migration resistance, and accelerate ion diffusion kinetics, ultimately delivering enhanced rate capability [58]. The topological defects induced by nanopore formation also markedly increase the effective SSA of the carbon matrix, offering more abundant active sites to further boost sodium storage capacity [59]. Zhao et al. further classified closed nanopores into two distinct categories based on gas permeability and electrolyte accessibility: fully closed pores (FCPs) that are impermeable to helium and semi-closed pores (SCPs) that allow helium penetration while blocking electrolyte access, with both categories retaining functionality as active sodium storage sites [60].

#### Defects

Defects also play a pivotal role in the structure of HC and are primarily categorized into intrinsic defects and extrinsic defects [57, 58]. Specifically, intrinsic defects sites can serve as adsorption centers for sodium ions, increasing the number of sodium storage sites [59]. In contrast, extrinsic defects mainly originate from the introduction of non-carbon atoms, such as oxygen-containing functional groups [60]. These functional groups not only enhance the surface activity of HC but also regulate its electronic structure, thereby influencing the adsorption strength and diffusion rate of Na^+^.

For intrinsic defect engineering, Yuan et al. have employed a metal-assisted catalytic strategy to synthesize a biomass-derived fibrous HC with high reversible intrinsic defects [61]. Catalytic tuning during thermal etching triggers *sp*^*2*^ hybridized carbon bonding reconstruction, generating low potential planar intrinsic defects at the expense of irreversible carbon edges. This configuration markedly enhances defect reversibility and interfacial charge transfer kinetics, boosting slope capacity within 0.1–1 V even at high current densities. Zhang et al. have argued that preventing the deactivation of intrinsic defects within closed nanopores during interfacial side reactions and activating them for reversible sodium storage are pivotal to enhancing sodium storage performance [57]. For extrinsic defect engineering, Zhang et al. developed a trace doping coupled defect modulation strategy, where 0.41 wt% phosphorus doping enables carbon matrix microstructure optimization via carbonization regulation, rather than acting as dominant active sites. The optimized PHC 1.5% anode delivers a reversible capacity of 364.98 mAh g^−1^, retaining 220 mAh g^−1^ even at 2 A g^−1^ [62].

As aforementioned, defects exert a dual effect on sodium storage in HC. Defects can serve as additional sodium storage sites and enhance the adsorption capacity for sodium ions, thereby boosting the sodium storage capability of HC [60–63]. Conversely, defects also give rise to dead sodium, which impedes the reversible release of sodium ions during charging–discharging processes and consequently reduces the ICE and cycling stability of HC [64]. Despite extensive advances in HC defect engineering, three core challenges remain unresolved. The structure activity relationship between defect types and sodium storage mechanisms remains unclear. The dynamic influence of defect concentration gradients on ion kinetics lacks direct in situ characterization evidence. The correlation between defect induced interphase evolution and long-term cycling stability requires further in-depth investigation.

#### Morphology

The morphological architecture of HC is a pivotal multiscale structural descriptor that dictates electrode processability, interfacial electrochemistry, and overall sodium storage performance. The prevailing particle geometries, including spherical, flake shaped, and irregular particles, impose distinct deterministic effects on sodium ion migration kinetics, solid electrolyte interphase (SEI) evolution behavior, and electrode–electrolyte interfacial compatibility, all of which are fundamental determinants of the practical electrochemical output of HC anodes. Comprehensive elucidation of morphology property correlations is therefore imperative to guide the rational structural design of HC materials tailored for targeted application scenarios.

Notably, spherical HC occupies a prominent position among these candidates owing to its unique geometric characteristics that synergistically address multiple critical challenges in sodium ion storage systems. The isotropic geometry of spherical HC particles minimizes the interparticle contact resistance and facilitates homogeneous electrolyte infiltration across the entire electrode matrix, which in turn expedites the charge transfer dynamics at the electrode–electrolyte interface and promotes rapid sodiation and desodiation processes [65]. Spherical HC typically features a reduced specific surface area compared with its flake counterparts. This structural merit effectively mitigates the excessive formation of irreversible solid electrolyte interphase layers during the initial cycles, thus elevating ICE as a key metric for evaluating the practical value of anodes [66]. Beyond this, the curved surface of spherical particles confers superior structural robustness, which enables effective accommodation of the volumetric fluctuation induced by repeated insertion and extraction of sodium ions [1–3]. Furthermore, the compact and ordered packing of spherical particles optimizes the electrode tap density, a core prerequisite for realizing high volumetric energy density in practical sodium-ion battery devices. Such a combination of electrochemical and structural merits renders spherical HC a highly competitive candidate for advanced SIBs. In contrast, flake-shaped HC boasts a core advantage in boosting ion diffusion kinetics; however, its low tapping density tends to compromise volumetric specific capacity, while aggregation issues induced by van der Waals forces between flakes necessitate mitigation via surface modification or porous structure engineering. Meanwhile, granular HC capitalizes on the versatility of its synthesis process and balanced electrochemical performance, holding promising potential for large-scale applications.

Spherical shape is not only beneficial for improving tap density and reducing side reactions, but also for forming high interface stability HC products through CVD coating in the later stage. Spherical HC is a better choice for CVD coating process due to its excellent fluidity, easier uniform coating and protection, and effective avoidance of engineering problems caused by flake particles. In CVD reactors, the mixing, transportation, and fluidization processes of spherical powders are extremely smooth, ensuring equal contact opportunities between gases (such as acetylene and methane) and each microsphere, laying the foundation for fluid mechanics to achieve uniform coating. The spherical HC surface has uniform curvature, isotropy, and low gas mass transfer resistance. The precursor gas can freely diffuse and react uniformly on the surface, causing the coating layer to grow isotropy and form a dense and uniform protective layer. The large size and high specific surface area of sheet-like particles make them more prone to particle adhesion, and the curvature differences on the surface are large, resulting in inconsistent deposition rates of precursor gases on the surface and difficulty in uniformly covering the coating layer. The spherical HC structure has no sharp edges, stress dispersion, and can effectively reduce the impact of collisions on the core, protecting the integrity of the original structure and coating layer. The sheet-like particle structure is fragile and prone to "corner wear" during motion and collision, resulting in local coating layer rupture and exposure of the core.

#### Contradictions and Synergies Between Microstructures of HC

**Fig. 3:**
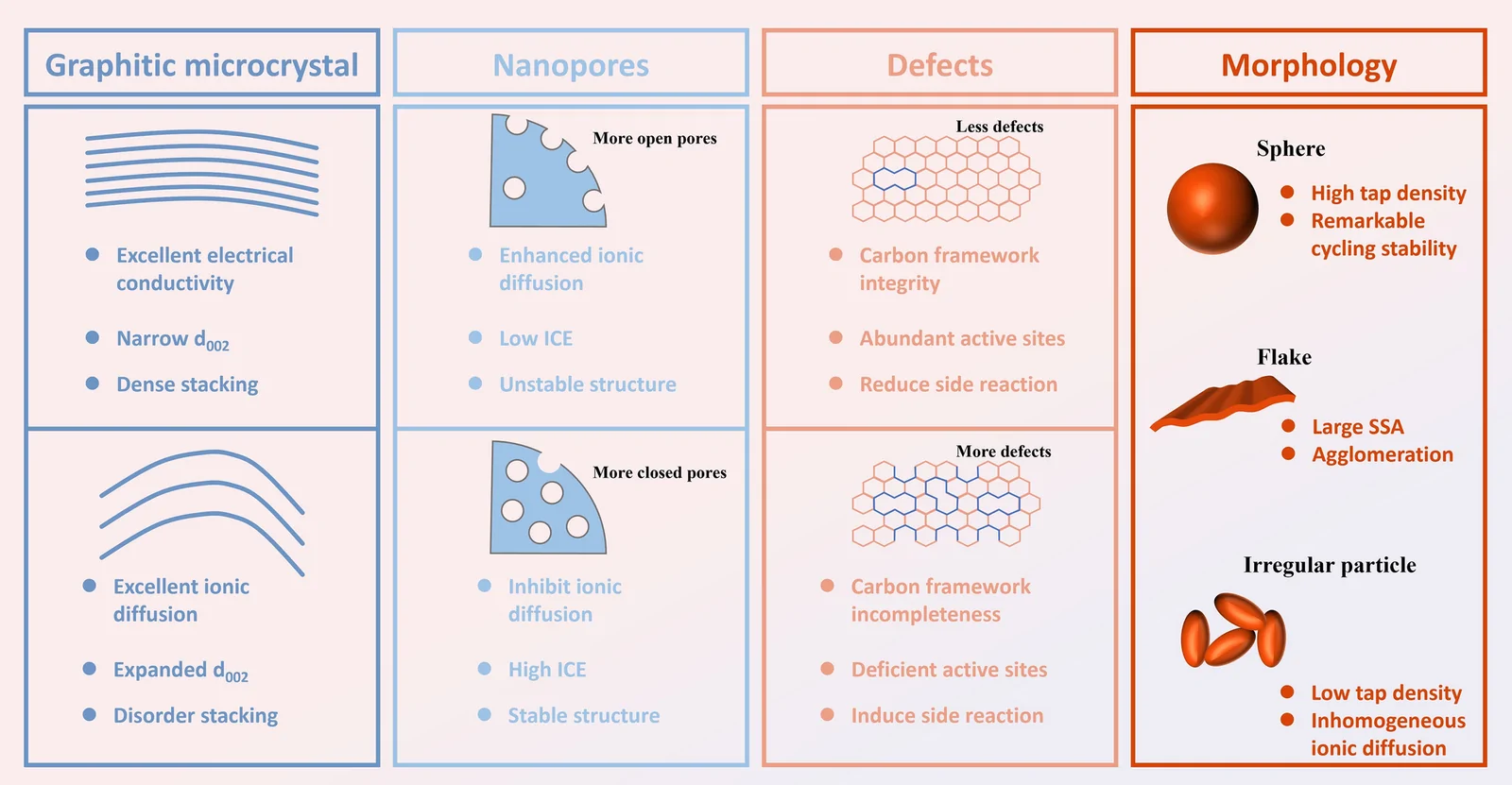
Contradictions and synergies between microstructures of HC

As shown in Fig. 3, the performance optimization of HC anode materials is fundamentally the result of achieving a dynamic balance among multiple structural contradictions at the atomic to nanoscale. These structural characteristics determine the performance of HC, including initial efficiency, rate capability, specific capacity, and cycling stability. Poor initial efficiency, poor rate capability, low volumetric specific capacity, and short cycling life are common issues with HC anode materials [9, 67, 68]. To address these issues, we need to conduct multi-scale design for HC.

ICE issue: The excessive specific surface area of HC, the presence of too many surface oxygen-containing functional groups (–COOH, –OH), and an abundance of defect sites all contribute to the irreversible decomposition of the electrolyte, leading to the formation of an excessively thick SEI film and subsequently reducing the ICE of HC. By depositing a dense, amorphous carbon layer on the surface of HC and within the pores through chemical vapor deposition (CVD) or liquid-phase coating, it is possible to physically shield the active sites and reduce electrolyte contact, thereby enhancing the ICE. Additionally, high-temperature treatment can facilitate graphitization and edge repair, eliminating dangling bonds and oxygen-containing groups at the edges, which can also improve the ICE.

Rate capability issue: Na^+^ has a relatively large radius, which results in slow diffusion between highly disordered carbon layers. An unreasonable pore structure can also lead to high migration barriers for ions, resulting in low-rate capability. Additionally, HC is composed of highly disordered and distorted carbon layers, which lack sufficient intrinsic electronic conductivity, further contributing to low-rate capability. Expanding the interlayer spacing is an effective method to increase the ion migration rate. Meanwhile, introducing atoms such as N and S into the carbon framework can create more defects and active sites, enhancing the adsorption capacity for ions, thereby improving capacity and rate capability. Optimizing the framework structure and constructing long-range graphite domains to enhance electron transport can also improve rate capability.

Cycling stability issue: Due to the large radius of sodium ions, when they are inserted into the nanopores and interlayers of HC, local volume expansion occurs. After repeated cycling, HC particles may develop microcracks and pulverization, leading to the destruction of the electrode structure and the fracture of the conductive network. The sodium storage capacity of HC primarily relies on its disordered structure and closed pores. During long-term cycling, the repeated stress of sodium ion insertion/extraction can cause the collapse of some pore walls or the destruction of the closed pore structure, leading to a reduction in effective sodium storage sites and rapid capacity fading. An unstable SEI film can cause continuous increases in interfacial impedance, continuous consumption of active sodium, and continuous increases in irreversible capacity. The sodium storage behavior in the plateau region is close to “intrapore condensation or quasi-metallic sodium” which is thermodynamically unstable and prone to agglomeration or side reactions with the electrolyte during cycling, resulting in a decrease in plateau capacity. Improving the surface properties of HC and forming a stable and uniform SEI film can enhance cycling stability. Meanwhile, optimizing the structure and chemical state of HC itself, such as constructing large interlayer spacing, isolated and disconnected closed pores, and low-barrier nucleation sites (doping, etc.), is beneficial for maintaining the structure of HC. A spherical structure is also conducive to achieving more uniform current distribution (uniform deposition) and stress release, thereby extending the cycling life.

Volume specific capacity issue: HC typically consists of irregular particles with low tap density (usually < 1.0 g cm^−3^), and a large number of ineffective open pores occupy the volume, all of which lead to a lower volume specific capacity. Preparing spherical HC is the most effective means to increase packing density. Furthermore, we believe that a bimodal or trimodal particle size distribution should be adopted, allowing small particles to fill the voids between large particles, which can increase the electrode compaction density by more than 20% without changing the material’s essence. At the same time, by regulating the pyrolysis behavior of the precursor, more effective closed pores can be generated. These strategies can all enhance the volume specific capacity.

**Fig. 4:**
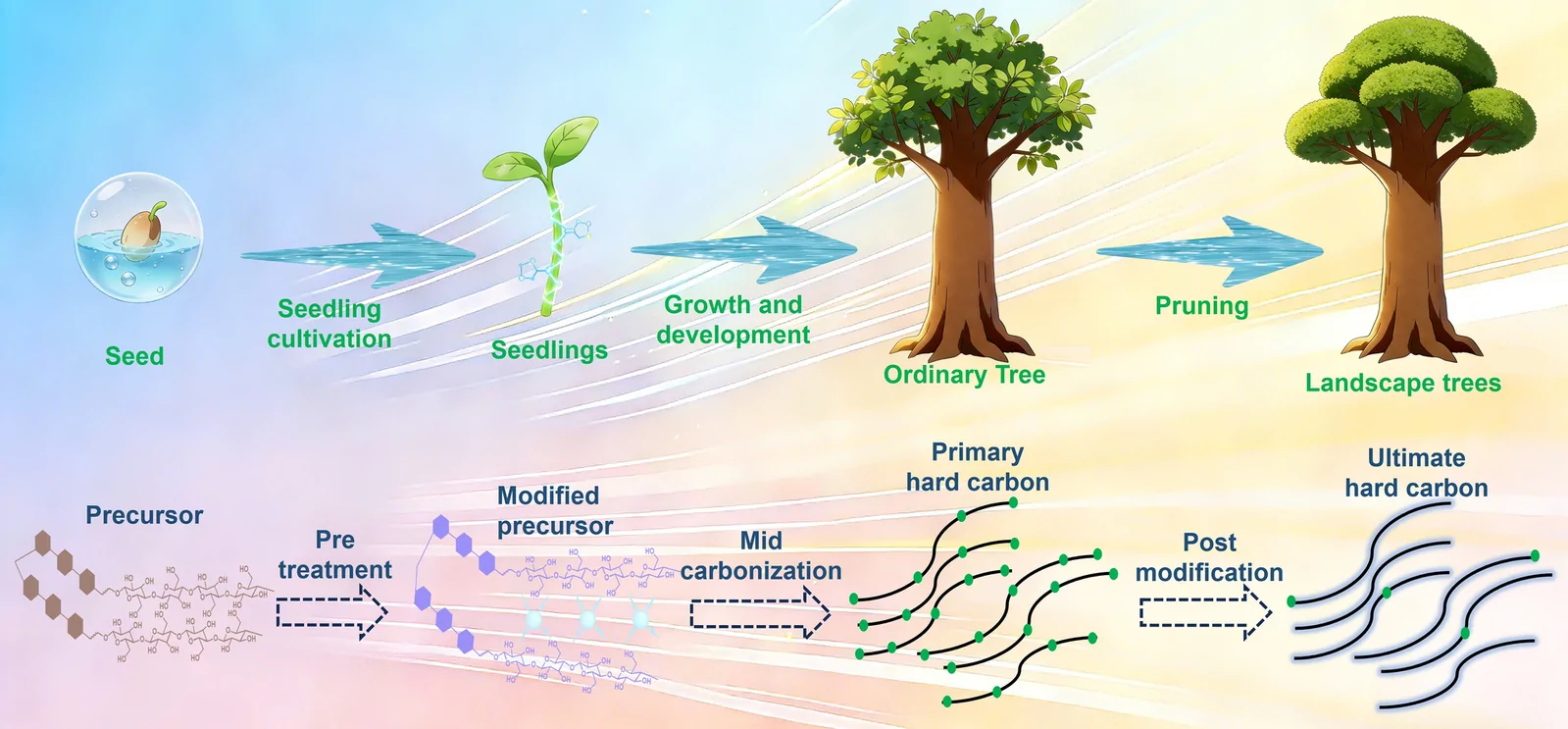
Rational selection of HC process routes for different precursors

The structural regulation of HC mentioned above is not isolated. For instance, doping, increasing interlayer spacing, and reducing microcrystallite size are necessary to enhance ion transport efficiency. However, this may lead to an increase in defects, a decrease in the orderliness of graphite domains, and a reduction in the conductivity and initial efficiency of HC. In structural tuning, there are both synergistic and antagonistic effects between crystallinity and disorder, closed pores and open pores, structural stability and ion accessibility, as well as between nanoscale particles and spherical micrometer-scale morphologies of HC. Therefore, the performance of HC cannot be achieved solely through linear adjustment of a single process parameter, but requires multi-stage and multi-scale collaborative precise control. As shown in Fig. 4, the holistic optimization for HC anode fabrication is analogous to the systematic cultivation of a high-quality landscape tree. The intrinsic physicochemical properties of the selected precursor define the fundamental performance ceiling of the final HC material, just as seed genetics determine a tree’s growth potential and stress resistance. The molecular composition and crosslinking density of the precursor inherently predetermine the nucleation tendency of graphitic domains and the initial pore-forming propensity during subsequent thermal treatment, laying the structural foundation for all downstream modification steps. Targeted pretreatment builds a robust, tunable foundation for downstream microstructural refinement, analogous to cultivating a resilient seedling root system and structural framework. Through molecular crosslinking, component regulation or defect pre-engineering, pretreatment modulates the thermal reactivity of the precursor matrix, which in turn dictates the growth kinetics of graphitic domains and the evolution of pore topology during carbonization. This stage preconfigures the spatial distribution of active sites for graphitic layer stacking and pore nucleation, effectively mitigating uncontrolled structural coarsening or pore collapse in subsequent high-temperature processing. Carbonization directly maps to the directional growth process of the tree. This stage forms the core structural framework of the HC material, just as the gradual growth of a tree constructs its trunk, branch network, and overall structural morphology. Controlled carbonization conditions govern the thermodynamic and kinetic pathways of carbonaceous rearrangement, which directly define the lateral size, stacking order and spatial distribution of turbostratic graphitic domains, alongside the size distribution, connectivity, and open–closed ratio of the pore network. These structural features formed in this stage establish the material’s intrinsic sodium storage framework, defining its core electrochemical properties including ion transport kinetics, electronic conductivity and long-term structural stability. Post-treatment corresponds to the precision pruning and shaping of mature timber. Just as targeted pruning removes redundant branches, optimizes structural morphology and enhances the ornamental and functional value of the landscape tree, post-treatment processes deliver targeted refinement of the preformed carbon framework. Post-treatment modulates the surface functional group of graphitic domains, passivate irreversible defect sites at pore edges, and realize targeted regulation of pore opening and closure without disrupting the integrity of the bulk carbon skeleton. Such modifications optimize surface physicochemical properties and improve interfacial compatibility, refining the material’s electrochemical performance to meet the stringent requirements of practical applications.

### Sodium Storage Mechanism

#### Typical Sodium Storage Mechanism Models

Typical static models, established on ex situ structural characterization and electrochemical performance correlation, form the foundational framework for understanding sodium storage in HC by attributing reversible capacity to discrete, steady-state behaviors matched with specific potential windows, without accounting for real-time structural evolution of the carbon matrix or dynamic sodium ion behaviors during cycling.

**Fig. 5:**
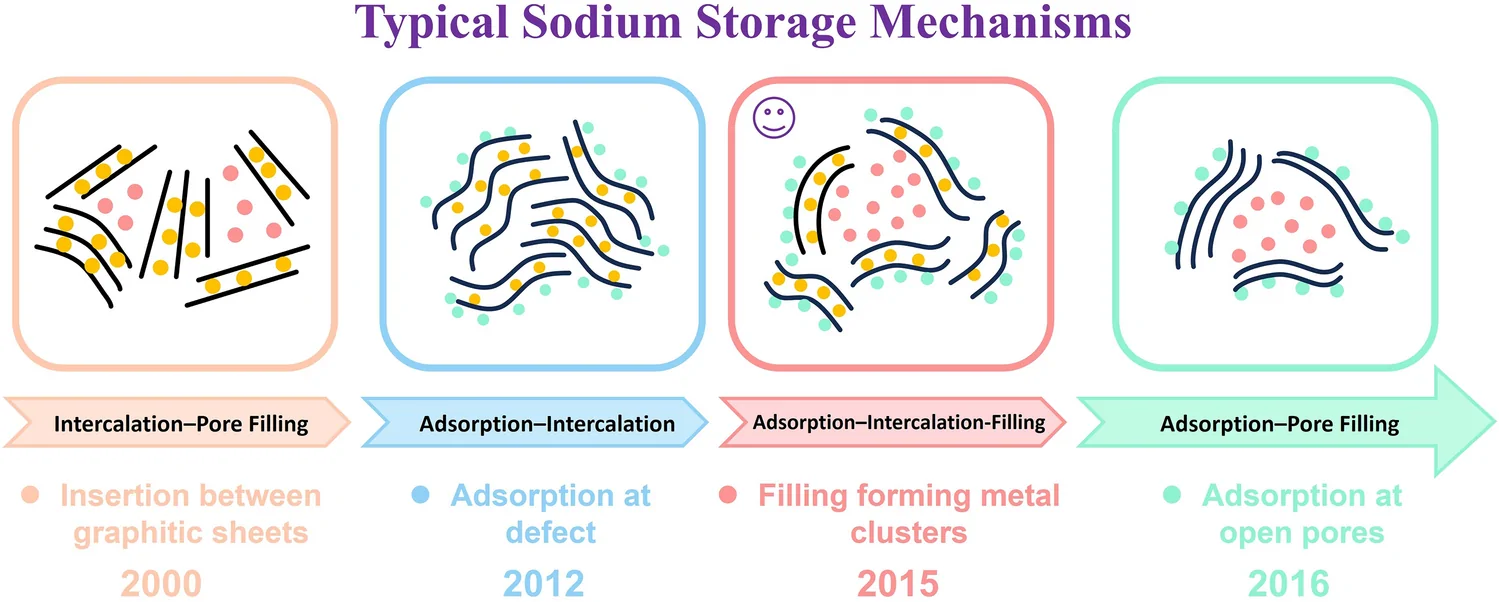
Typical sodium storage mechanism

As shown in Fig. 5, the pioneering intercalation-filling model, proposed by Stevens and Dahn in 2000, first deconstructed sodium storage into two sequential steps: sodium intercalation into expanded graphitic interlayers of turbostratic nanodomains in the sloping region, followed by quasi-metallic sodium filling into closed nanopores in the low-voltage plateau region [21]. This model laid the cornerstone for subsequent research by correlating microstructural features with electrochemical output, yet overlooked defect-derived capacity and failed to interpret the performance of HC with negligible interlayer expansion or closed porosity. To address these limitations, the adsorption-filling model was put forward, supported by in situ X-ray diffraction observations of unshifted (002) peaks during sodiation in the sloping region [67]. This model excluded intercalation entirely, ascribing sloping capacity exclusively to reversible sodium adsorption on surface/edge defects, heteroatom dopants, and pore walls, while retaining the quasi-metallic pore-filling mechanism for the plateau region. This framework explained the high sloping capacity of defect-rich HC, but could not account for the universal capacity enhancement from expanded graphitic interlayer spacing. For highly graphitized HC with minimal closed porosity, the adsorption–intercalation model was developed, which eliminated pore-filling entirely and attributed total capacity solely to sodium adsorption on defect sites in the high-potential region and intercalation into expanded graphitic interlayers in the mid-potential region [9]. This model matched the electrochemical behavior of low-porosity HC, but failed to explain the well-documented linear correlation between closed pore volume and plateau capacity. With accumulated multi-scale characterization and electrochemical validation, the three-stage adsorption–intercalation–filling collaborative model has emerged as the most widely accepted consensus framework [68]. This model integrates the merits of prior frameworks, dividing sodium storage into three potential-correlated steady-state stages: sodium adsorption on surface/defect sites with partial intercalation into expanded interlayers in the high-potential sloping region, quasi-metallic filling into closed nanopores in the low-voltage plateau region, and surface sodium deposition in the ultra-low potential region with inherent dendrite risks [69].

Despite their widespread adoption, all classic static models share critical intrinsic limitations. These models only provide macroscopic qualitative descriptions of sodium storage behaviors corresponding to potential windows and cannot define, at the molecular scale, the temporal evolution sequence of sodium intercalation and pore-filling, the spatial distribution of precise active sites, or the real-time migration path of sodium ions within the carbon matrix. Furthermore, these models treat distinct sodium storage behaviors as independent linear superposition processes, neglecting coupling effects and dynamic competition between different behaviors, which explains their inability to resolve widely observed experimental phenomena including potential hysteresis, nonlinear kinetic evolution, and performance mismatches between half-cell and full-cell systems.

#### Advanced Dynamic Sodium Storage Mechanisms

**Fig. 6:**
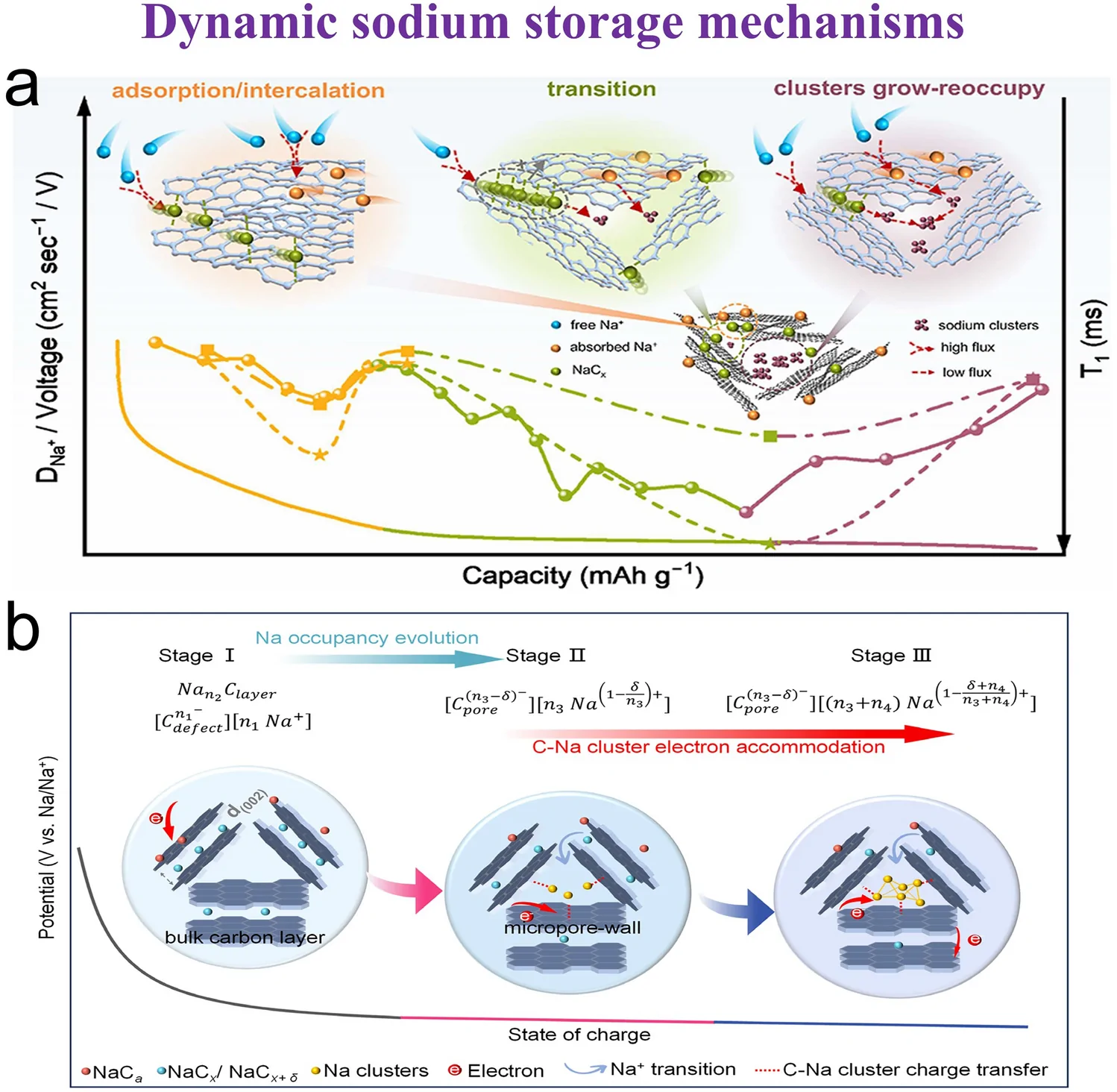
**a**, **b** Dynamic sodium storage mechanisms [71, Copyright 2025, American Chemical Society. 72, Copyright 2026, Wiley.]

Recent advances in state of the art in situ and operando characterization methodologies have enabled unprecedented molecular level resolution of sodium storage behaviors in HC, overturning the view of static discrete reaction pathways and confirming the highly dynamic nature of sodiation and desodiation processes. As shown in Fig. 6, based on the three-stage sodium storage theory, some more specific dynamic models have been proposed [70, 71].

As shown in Fig. 6a, Li et al. proposed a “sodium release-transformation non-equilibrium path” model, deepening the understanding of the three-stage mechanism in 2025 [70]. Stage 1, corresponding to the sloping region of 1.5–0.15 V, features sodium storage dominated by the intercalation of sodium ions into graphitic interlayers to form NaC_x_, accompanied by simultaneous ion adsorption at defect sites. Stage 2, covering the early plateau region of 0.15–0.05 V, acts as the critical transition period. Sodium species previously accommodated via intercalation and adsorption are released and transformed into quasi-metallic sodium clusters confined within closed nanopores. A sharp decline in sodium ion diffusion coefficient occurs during this process, which constitutes the kinetic bottleneck of the overall sodium storage reaction. This evolution of diffusion kinetics is strongly correlated with the cluster formation process and acts as a key determinant of the rate capability of HC electrodes. Stage 3, corresponding to the late plateau region of 0.05–0 V, involves continuous growth of sodium clusters within the closed nanopores, alongside simultaneous re-adsorption and re-intercalation of sodium ions at the newly liberated defect sites and interlayer galleries.

As shown in Fig. 6b, Zhong et al. advanced the mechanistic understanding by proposing a three-stage sodium storage model governed by “electronic structure evolution” rather than pore volume alone in 2026 [71]. Using operando ^23^Na NMR spectroscopy, they quantitatively tracked the evolution of sodium occupancy at distinct molecular level sites in HC, including interlayers within graphite type domains, sites associated with defects, intercalation states with high sodium concentration, and quasi-metallic sodium clusters confined within micropores. They identified a distinct transition in sodium occupancy in the early plateau region of 0.05–0.2 V, where sodium migrates from graphite type interlayers and defect associated sites to form quasi-metallic sodium clusters within the micropores. Complementary ^13^C NMR spectroscopy furtherly proved that carbon layers at the micropore walls act as electron acceptors, undergoing charge transfer with sodium clusters to fill the π* orbitals and reduce the aromaticity of the carbon framework. Charge localization occurs exclusively at the micropore walls rather than the bulk carbon layers, a feature that accounts for the stabilization of quasi-metallic sodium clusters via the overlap of surface electric fields. Based on these molecular level insights, the full sodium storage process is delineated into three sequential stages tightly coupled with electronic structure evolution. In the initial Stage I corresponding to the sloping region, sodium intercalates into graphite type layers and adsorbs at defect sites, accompanied by electron injection into the carbon matrix and a sloped decrease in cell potential. Following this, in Stage II covering the early plateau region, quasi-metallic sodium clusters form within the micropores, with carbon layers at the micropore walls participating in charge transfer. The migration of sodium from interlayer and defect sites to the micropores results in a marked decrease in sodium ion diffusion coefficient. In the final Stage III corresponding to the late plateau region, electrons are predominantly injected into sodium clusters and bulk carbon layers, while the electronic environment of the micropore walls remains stable. The graphite-rich HC sample exhibits an inflection in potential due to its limited micropore volume, which corresponds to the re-intercalation of sodium into graphite type layers, whereas the defect-rich HC sample maintains continuous pore filling throughout this stage.

Despite these significant advances in molecular-level resolution of dynamic sodium storage mechanisms, several core controversies remain unresolved within the academic community. First, the exact physicochemical state of sodium accommodated within closed nanopores, whether quasi-metallic or ionic, remains a subject of ongoing debate. Second, a fully quantitative correlation between pore size distribution and plateau capacity has yet to be established, limiting the predictive design of HC microstructures. Third, the dynamic evolution of sodium storage mechanisms during long-term cycling remains poorly understood, which hinders the development of HC anodes with extended cycle life. Resolving these controversies is critical to guiding the rational microstructure design of high-performance HC anodes and represents a key priority for future research in this field.

## Pre-treatment

HC pretreatment technology centers on the regulation of the molecular structure and micro nano-morphology of carbon precursors [72–74]. Based on the differences in modification mechanisms and process characteristics, pretreatment technologies can be systematically categorized into these types, including impurity treatment, hydrothermal treatment, chemical crosslinking, pre-carbonization, pre-oxidation, pre-doping, component regulation, and pore-forming treatment. Each strategy achieves precise regulation of the bulk structure of HC through unique physicochemical interaction mechanisms.

### Crosslinking

#### Hydrothermal Crosslinking

**Fig. 7:**
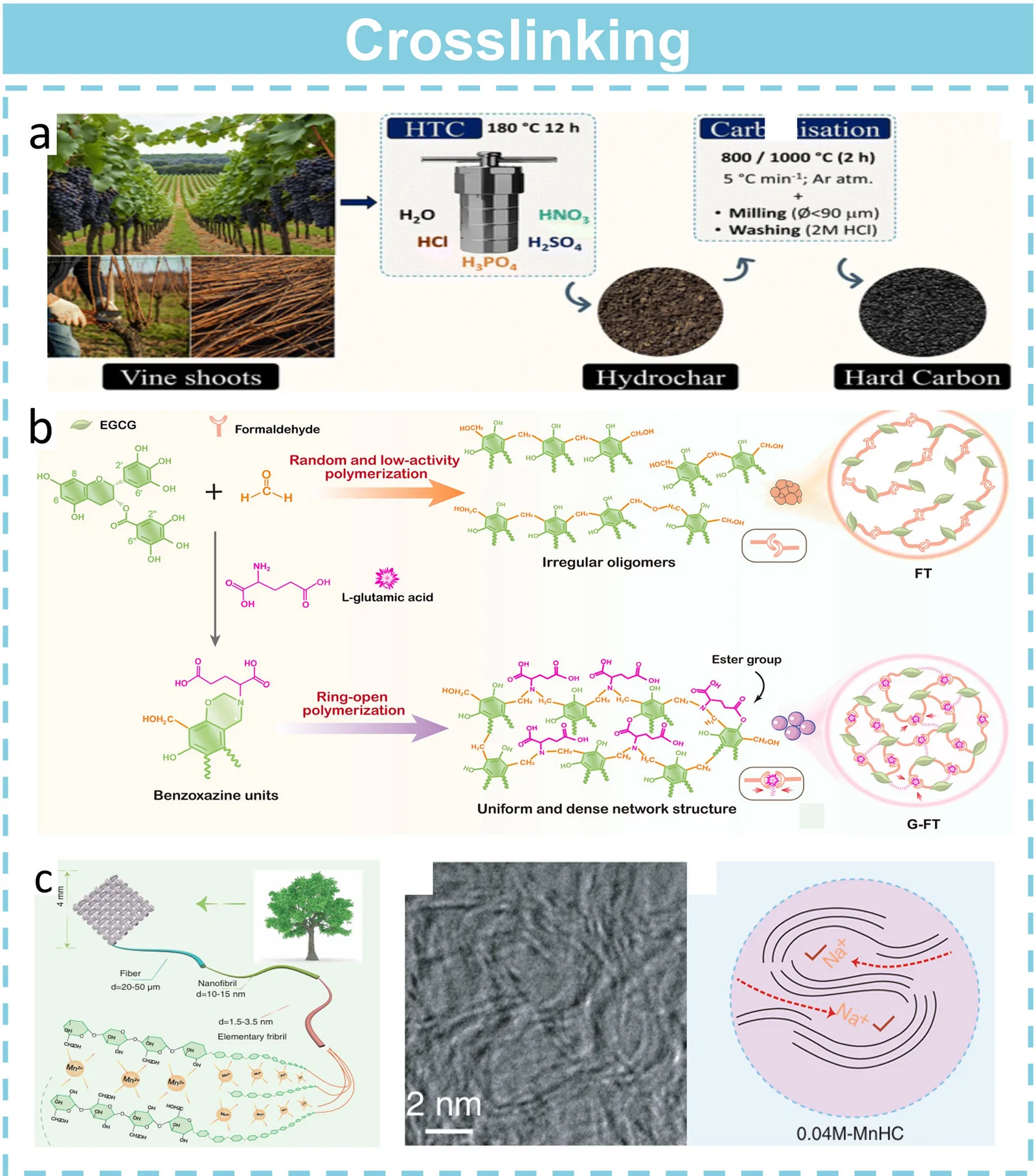
**a** Acid-assisted hydrothermal synthesis strategy for HC [81]. Copyright 2025, The Royal Society of Chemistry. **b** Benzoxazine chemical directed cross-linking [82]. Copyright 2026, Wiley. **c** Mn^2+^ crosslinking strategy [88]. Copyright 2023, Wiley

Hydrothermal treatment regulates the morphology and microstructure during the formation of HC materials. The smooth spherical morphology induced by this process results in high packing density, which helps shorten the diffusion paths of ions and electrons. For biomass precursors such as lignin, cellulose, and starch, hydrothermal treatment can disrupt their complex natural structures and modify their component content [75]. Meanwhile, it can remove partial impurities and volatile components from precursors, enhance the thermal stability and reactivity of precursors, generate intermediate products favorable for carbonization, and ultimately improve the carbonization yield and properties of HC materials [76, 77]. In a representative study, Ma et al. adopt hydrothermal carbonization as a tunable pretreatment process for oak leaf OL biomass waste precursor [78]. This approach achieves a smooth spherical morphology with high bulk density 0.72 g mL^−1^, expanded layered graphitic domains d_002_ ≈ 0.394 nm, and well-developed micro-mesoporous structures. It also introduces additional active sites, including defects and nano-vacancies, which collectively enhance the overall sodium storage performance. Maria-Magdalena Titirici et al. have synthesized a series of HC with curved short-range graphitic domains [79]. They achieve precise regulation of the d_002_ from 0.355 to 0.410 nm and optimization of the ID/IG ratio from 1.18 to 0.88. Among these materials, the optimal sample G1500 exhibits the highest reversible capacity of over 340 mA h g^−1^ at a current density of 30 mA g^−1^, though its rate capability still requires further improvement. In a separate study, the merits of spherical morphology and hierarchical pore architecture for uniform ion flux, as well as the pivotal role of surface oxygen-containing groups in achieving homogeneous interfacial properties, are demonstrated [80]. Alvira et al. have utilized acids (e.g., HNO₃, HCl) as catalysts to drive the degradation of grapevine branches under hydrothermal conditions of 180 °C for 12h (Fig. 7a) [81]. This process synergistically introduces oxygen-containing functional groups while tailoring the morphology, pore structure, and surface chemical properties of the resultant material, ultimately yielding HC with enhanced sodium storage.

Despite these merits, the efficacy of hydrothermal treatment is comprehensively influenced by multiple factors, including temperature, time, pH, reactant concentration, and reaction medium. Meanwhile, the hydrothermal treatment imposes certain requirements on the properties of raw materials, with different raw materials exhibiting significant differences in their behavior during hydrothermal reactions and in the structure of products [82]. For some specific raw materials, a hydrothermal treatment may fail to achieve the desired effects, requiring pretreatment or the selection of alternative modification methods. The interactions between these factors are complex, and extensive experimental optimization is required to determine the optimal processing conditions. Hydrothermal treatments simultaneously modify the morphology, introduce oxygen functionalities, and remove impurities. However, its effectiveness is a classic multivariate optimization problem, heavily dependent on precursor nature and processing parameters. The spherical morphology may come at the expense of a reduced specific surface area for ion adsorption, and the introduced oxygen groups, while beneficial for wettability and large interlayer spacing, can exacerbate ICE loss.

Therefore, it is best viewed as a flexible preconditioning step, particularly suited for biomass, and success must be validated by subsequent carbonization stability. The spherical morphology, crosslinking density, and surface functional group composition modulated via hydrothermal treatment directly define the feasible operating window of subsequent carbonization processes. Hydrothermally synthesized carbonaceous spheres with high crosslinking density exhibit exceptional thermal stability, enabling the use of faster heating rates and higher terminal carbonization temperatures without structural collapse, in contrast to unmodified biomass precursors that require slow, staged heating to avoid pore structure degradation. Meanwhile, the hierarchical pore framework pre-constructed via hydrothermal treatment directly governs the effectiveness of downstream post-treatment processes. The three-dimensionally interconnected mesopores formed during hydrothermal treatment facilitate uniform infiltration of liquid-phase doping reagents and homogeneous gas-phase deposition during CVD post-treatment, eliminating the issue of uneven surface modification prevalent in dense, non-porous HC matrices. Furthermore, hydrothermal pretreatment can reduce the reliance on remedial post-treatment modifications. The introduction of nitrogen-containing functional groups during hydrothermal treatment enables in situ bulk heteroatom doping during subsequent carbonization, avoiding the structural damage and inhomogeneous doping associated with post-treatment liquid-phase doping.

#### Chemical Crosslinking

The chemical crosslinking strategy enables efficient regulation of the pore evolution behavior of carbon matrices during pyrolysis via the introduction of chemical crosslinking agents into precursor systems to establish three-dimensional network structures. These pore structures, encompassing micropores, mesopores, and macropores, create favorable conditions for sodium ion storage and transport. Such crosslinking-induced topological confinement effect can enhance the proportion of *sp*^2^ hybridized carbon domains in HC materials [83].

1. Covalent crosslinking
  Covalent crosslinking induces the formation of stable covalent bond networks between precursor molecular chains via the introduction of crosslinking agents such as aldehydes, acid anhydrides, or epoxies, thereby regulating the thermodynamic behavior and structural evolution during carbonization. Ma et al. have treated semicoke with citric acid as a crosslinking agent, converting the semicoke from a thermoplastic precursor to a thermosetting one [84]. The –C–(O)–O– groups effectively inhibit the rearrangement of carbon microcrystals in semicoke during carbonization, resulting in the formation of abundant pseudo-graphitic structures, larger carbon interlayer spacing, and micropores. The optimized OHC-2 electrode exhibits a high ICE of 81% and a specific capacity of 307 mAh g^−1^. Compared with unmodified R-HC, its low-voltage plateau capacity and sloping capacity increase by 2.5 times and 1.7 times, respectively. Another study by Wang et al. uses polyether amine d-2000 (PEA) to form helically arranged micelles [85]. Glucose molecules are anchored on the micelle surface via intermolecular hydrogen bonds, forming core–shell structured composite micelles (PEA-X/Glu). During the high-temperature carbonization stage, PEA is removed under a nitrogen atmosphere, which converts the ordered carbon skeleton obtained by carbonization into wrinkled HC materials with high and controllable crystallinity. Wu et al. leveraged the multiple reactive site ligation mechanism of glutamic acid to concurrently trigger in situ Mannich and esterification reactions between the amino acid and tea polyphenols, thereby constructing a highly cross-linked framework with distinctive Mannich bridging motifs (Fig. 7b) [82].
  Additionally, Liu et al. have used phytic acid as a modifier and produced In situ phosphorus-doped spherical HC through the crosslinking between glucose and phytic acid and repairing some defects in the carbon layers [86]. Upon increasing the phytic acid content, TEM validates that the d_002_ interlayer spacing of the materials expands from 0.378 to 0.382 nm. The I_D_/I_G_ ratio increases from 1.006 to 1.050, effectively enhancing the carbon layer defect density, which is beneficial for Na^+^ storage and transport. The optimized sample PHC-0.2 shows an enhanced reversible capacity of 343 mAh g^−1^ at 20 mA g^−1^.

(2) Coordination crosslinking
  Coordination interactions between transition metal ions (e.g., Fe^3^⁺, Al^3^⁺) and oxygen/nitrogen-containing functional groups are utilized to construct spatially confined cross-linked networks, enabling the simultaneous regulation of pore structures and electron transport properties. Ji et al. have reacted zinc nitrate with gelatin, where numerous functional groups (–COOH and –NH_2_) in collagen molecules chemically anchor and monodisperse zinc ions to form coordination bonds [87]. This approach allows rational control over defect content and pore structure, thereby tuning the electrochemical performance of the material. The optimized HC (HC) exhibits a high reversible capacity of 400 mAh g^−1^ and excellent structural stability over 10,000 cycles, with a capacity retention of 77.8%. Zhao et al. have leveraged the coordination effect between manganese ions and oxygen-containing defects to catalyze the growth and rearrangement of graphitic domains in HC, resulting in the formation of nanoscale graphitic domains and carbon micro-pores (Fig. 7c) [88]. The optimized 0.04 M-MnHC electrode shows a reversible capacity of 336.8 mAh g^−1^ at 20 mA g^−1^ and a high ICE of 92.05%. Hou et al. have leveraged the synergistic effect of zinc oxalate and aminophenol to synergistically modulate the microstructure of resin-based HC [89]. Specifically, the chemical coordination of cations facilitates the formation of open pores via etching, while the crosslinking interaction between resin carboxyl groups and phenolic hydroxyl groups inhibits the rearrangement of graphite sheets and drives the bending of carbon layers surrounding nanopores, thus realizing the conversion of open pores to closed ones.
  However, residual unreacted crosslinking agents (approximately 0.8–1.2 wt%) during the chemical crosslinking process may block partial mesoporous channels, impeding the transport and diffusion of sodium ions. Furthermore, thermal decomposition of crosslinking agents may introduce heteroatoms such as O and N. Although these heteroatoms can enhance surface wettability, they exacerbate the uneven growth of the SEI, impairing the ICE and thus limiting the material’s practical application.
  A highly cross-linked thermosetting precursor network inhibits volatile release and molecular rearrangement during pyrolysis, enabling the use of rapid heating rates without severe pore structure collapse, while lightly cross-linked thermoplastic precursors require slow, staged low-temperature pyrolysis to avoid excessive fusion and pore closure. Crosslinking agents with heteroatom-containing functional groups enable in situ doping of the carbon matrix during pyrolysis, which can reduce or even eliminate the need for post-treatment heteroatom doping to modulate electronic conductivity.

#### Pre-carbonization

The purpose of pre-carbonization is to achieve the decomposition and reorganization of precursors by controlling pyrolysis conditions, thereby laying the foundation for subsequent carbonization and graphitization processes. In the preparation of HC materials, pre-carbonization can effectively regulate the microstructure and chemical composition of HC, which in turn influences its sodium storage performance. Through pre-carbonization, partial volatile components in the precursor can be removed, reducing the impurity content in HC materials, enhancing the purity and structural stability of the material, and modulating the interlayer spacing and pore structure of HC [90–92].

**Fig. 8:**
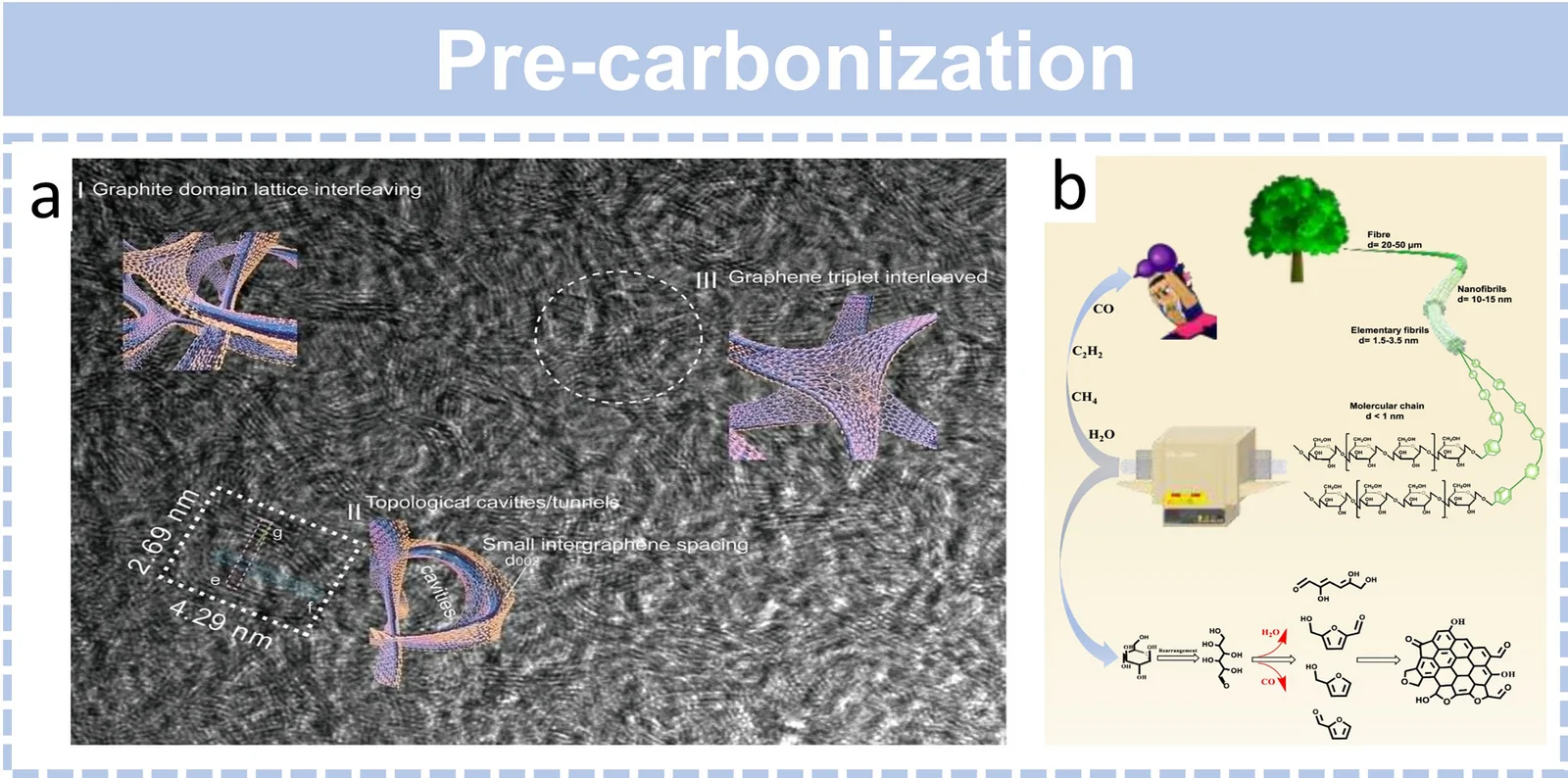
**a** Pre-carbonization mediated highly topological graphitized HC structure [93]. Copyright 2023, Wiley. **b** Vacuum pre-carbonization strategy and corresponding structural evolution [96]. Copyright 2025, Elsevier

Pre-carbonization can alter the molecular structure and chemical bonding state of precursors and provide more favorable conditions for subsequent carbonization reactions. Wang et al. have utilized corn cobs as the raw material [93]. By controlling the heating rate of pre-carbonization and carbonization temperature, HC precursors with varying crosslinking defects are prepared (Fig. 8a). A second-step high-temperature treatment then forms thin, twisted, and highly topological graphitized carbon (H-TPGC). This thin and twist-interleaved structure significantly reduces structural strain during the reversible insertion/extraction of sodium ions, thereby enabling a sodium ion battery with full plateau capacity. H-TPGC exhibits a plateau capacity of 290 mAh g^−1^, accounting for nearly 97% of the total capacity. Subsequently, Li et al. have employed almond shells as the precursor and adopted a two-step carbonization process involving pre-carbonization and high-temperature heat treatment [94]. This process yields the A-2.25-6-T electrode featuring a large interlayer spacing (0.389 nm) and long graphitized domains (*La* = 10.91 nm, *Lc* = 1.75 nm). At a current density of 10 A^−1^, the anode shows a reversible capacity of 128.3 mAh g^−1^. Deng et al. have used peeled pine wood as the raw material [95]. By means of a pre-carbonization process at 500 °C and an ensuing high-temperature treatment, they promote micropore closure, with the interlayer spacing decreasing from 0.389 nm to 0.384 nm. XPS discloses that the material exhibits a higher I_D_/I_G_ ratio after pre-carbonization, and the graphitized domains evolve into a growing disordered structure. Huang et al. have utilized cellulose as the precursor to precisely regulate the vacuum pressure at a controlled pressure of 0.06 MPa and engineer hierarchical open pore channels (Fig. 8b) [96]. Subsequent high-temperature carbonization facilitates the formation of closed pore nanostructures, reduces the oxygen concentration, and modulates the graphitization degree. XRD shows that the (002) peak shifts to the left with elevated vacuum degree, and the proportion of ordered graphitic regions increases from 42.3% to 54.3%, confirming that vacuum conditions promote carbon layer rearrangement. Raman scattering shows that the ID/IG ratio decreases from 2.18 to 1.81, while the *La* value increases from 8.82 to 10.62 nm, manifesting a reduction in carbon structural defects. Zhou et al. have pre-carbonized waste olive shells at 300 °C under three distinct atmospheres (air, argon, and argon–hydrogen mixture), followed by uniform high-temperature treatment under argon to prepare HC samples with atmosphere-dependent pre-carbonization [97]. The OSHC-Ar/H_2_ sample (pre-carbonized under argon–hydrogen mixture) possesses more pores, defects, and shorter graphitized domains than the OSHC-Ar sample (pre-carbonized under argon).

The pre-carbonization temperature and holding time are directly dictated by the physicochemical properties of the precursor modulated via upstream pretreatment, including component regulation, pre-oxidation, and chemical crosslinking. Pre-oxidized precursors with high oxygen content require lower pre-carbonization temperatures to avoid excessive crosslinking and pore shrinkage, while deashed biomass precursors with high cellulose content demand higher pre-carbonization temperatures to establish a stable carbon skeleton prior to high-temperature treatment. Meanwhile, the pre-carbonization process directly defines the optimal parameter window for subsequent high-temperature carbonization. Furthermore, pre-carbonization directly modulates the demand for downstream post-treatment modifications. The controlled defect formation and pore structure stabilization achieved via pre-carbonization reduce the formation of irreversible surface defects during subsequent high-temperature carbonization, minimizing the need for post-treatment surface coating and defect passivation to enhance ICE. In turn, the selected high-temperature carbonization technology imposes strict constraints on pre-carbonization protocol design. For flash Joule heating, pre-carbonization is a mandatory prerequisite to enhance the electrical conductivity of the precursor, ensuring uniform ultrafast heating across the material bulk. This coordinating role confirms that pre-carbonization cannot be designed as an isolated thermal treatment step, but must be fully integrated with both upstream precursor modification and downstream pyrolysis and post-treatment processes.

#### Pre-Oxidation

Pre-oxidation refers to the process of subjecting precursors to oxidation at relatively low temperatures to initially convert them into oxidized carbonaceous materials during the preparation of HC [98, 99]. This process is typically conducted under an oxidizing reagent or oxygen atmosphere to facilitate the oxidation reaction of the precursor. The key reasons for pre-oxidation of different raw materials are different [98]. The oxidation of coal and asphalt raw materials is mainly to induce more cross-linking and form a HC structure, while biomass oxidation is more aimed at optimizing the pyrolysis process and thus optimizing the HC structure.

**Fig. 9:**
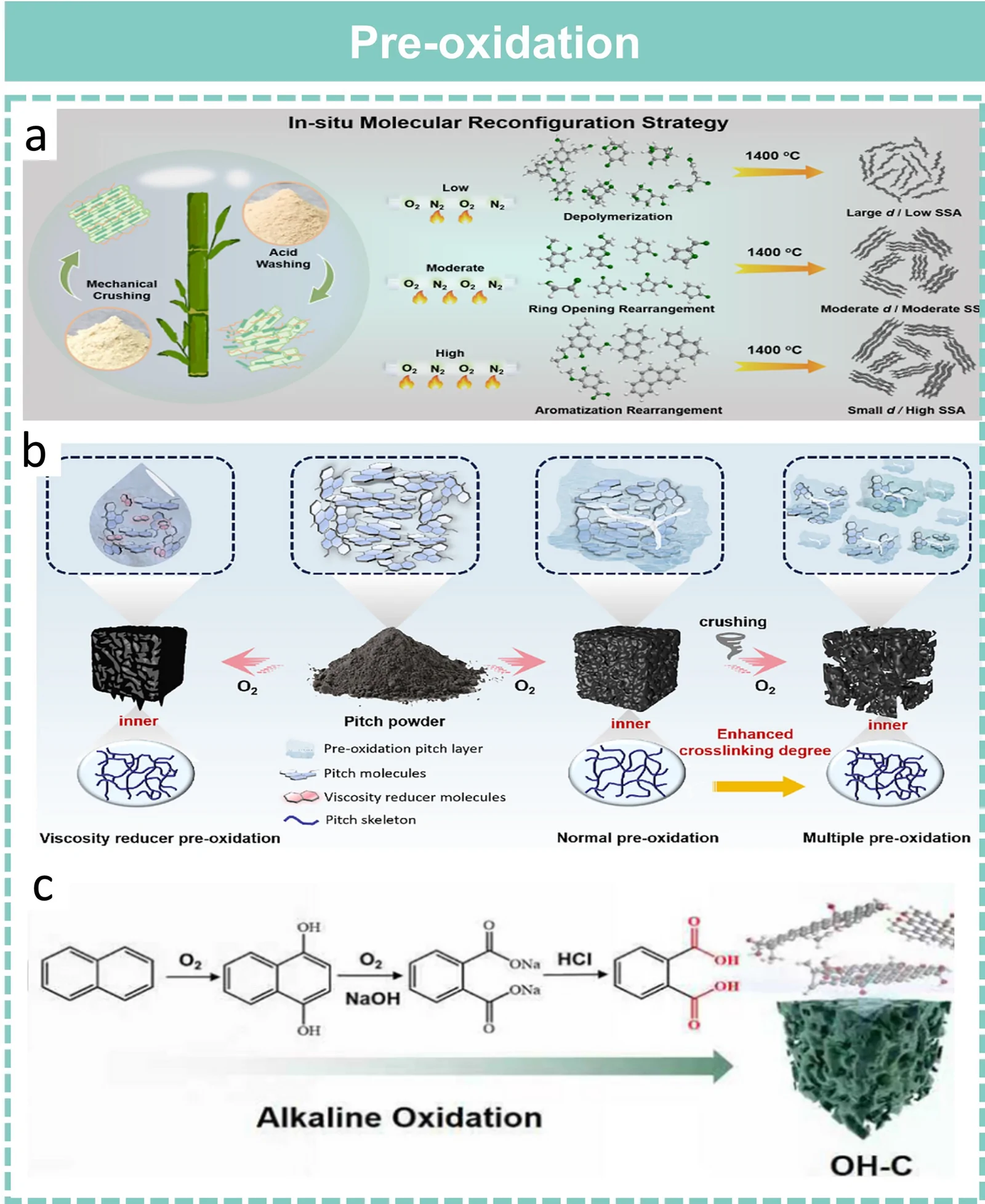
**a** Effects of different pre-oxidation temperatures on HC structure [102]. Copyright 2025, Wiley. **b** Schematics of different pre-oxidation processes, corresponding functional groups, defects, and HR-TEM [104]. Copyright 2025, The Royal Society of Chemistry. **c** Alkali-oxygen oxidation strategy [106]. Copyright 2025, Wiley

1. Biomass precursors
  Biomass derived precursors, including lignocellulosic biomass, agricultural and forestry wastes, and saccharide-based feedstocks, are inherently rich in oxygen containing functional groups such as hydroxyl, carboxyl, and carbonyl moieties, alongside a complex macromolecular framework with abundant reactive sites. For this class of precursors, the primary objective of pre-oxidation is not the introduction of additional oxygen containing groups, but the targeted regulation of the type, distribution, and crosslinking activity of inherent oxygen containing moieties [99]. Mild pre-oxidation eliminates excessive labile oxygen containing groups that would otherwise trigger violent volatile release and severe pore structure collapse during high-temperature carbonization, while converting unstable terminal oxygen groups into stable bridging oxygen containing functional groups [100]. These stable bridging moieties act as crosslinking nodes to construct a robust thermosetting network prior to carbonization, which effectively inhibits the random stacking of graphitic layers during pyrolysis and ensures homogeneous evolution of the pore structure. The secondary regulatory effect of pre-oxidation for biomass precursors lies in the homogenization of the precursor matrix, which mitigates the component heterogeneity inherent to natural biomass feedstocks and improves batch to batch consistency of the resultant HC. Excessive oxidation for biomass precursors must be strictly avoided, as it will introduce a high density of irreversible oxygen containing defects, leading to severe parasitic side reactions and reduced ICE of the final anode material [101, 102]. Wu et al. have precisely controlled the degradation sequence of cellulose, hemicellulose, and lignin in bamboo powder by regulating pre-oxidation temperatures (275–375 °C), thereby optimizing the crosslinking degree of carbon precursors (Fig. 9a) [102]. After secondary high-temperature carbonization, gradient structured HC is obtained. Specifically, a low pre-oxidation temperature (HC-AL) produces highly disordered layers (interlayer spacing of 0.417 nm, abundant micropores), while a high pre-oxidation temperature (HC-AH) generates graphitoid long-range ordered structures (interlayer spacing of 0.371 nm). Mao et al. have investigated the effect of air pre-oxidation on the crystalline structure and cross-linked structure of cellulose macromolecules [103]. Pre-oxidation can disrupt the hydrogen bond network of cellulose in advance and release a large number of active hydroxyl groups on the surface, which are then oxidized to form ether and ester crosslinking bonds. Ether bonds could cross-link and extend carbon layers horizontally, with the curved carbon layers encapsulating a well-developed interconnected pore structure. The HC prepared by pre-oxidation at 300 °C for 12h shows a specific capacity of 335 mAh g^−1^ and an ICE of 89%.

(2) Fossil fuel precursors
  Petroleum-derived precursors such as coal tar pitch, petroleum pitch, and aromatic resins and coal-derived precursors including anthracite, bituminous coal, and lignite feature a highly condensed aromatic macromolecular framework with extremely low inherent oxygen content and minimal reactive functional groups [104–106]. For this class of precursors, the core objective of pre-oxidation is the targeted introduction of oxygen containing functional groups onto the aromatic molecular chains, to construct covalent crosslinking bridges between adjacent aromatic domains and tailor the interlayer spacing of the resultant HC. During pre-oxidation, oxygen molecules attack the active sites at the edges of aromatic lamellae, introducing carboxyl, carbonyl, and hydroxyl groups onto the macromolecular chains. This newly introduced oxygen containing moieties triggers dehydration condensation and crosslinking reactions between adjacent aromatic molecules at elevated temperatures, forming a three-dimensional thermosetting cross-linked network. This cross-linked network effectively inhibits the melting and flow of thermoplastic pitch and coal precursors during the early stage of carbonization, preventing the excessive stacking and densification of aromatic lamellae that would otherwise lead to contracted interlayer spacing. Through precise control of oxidation degree, the interlayer spacing of the final HC can be steadily expanded, which provides more active sites for reversible sodium ion intercalation and enhances the rate capability of the anode. Unlike biomass precursors, moderate deep oxidation is often required for petroleum and coal derived precursors to achieve sufficient crosslinking density, as their inherent reactive sites are extremely limited. Insufficient oxidation will fail to construct an effective crosslinked network, leading to severe phase separation and structural inhomogeneity during high-temperature pyrolysis.
  Hu et al. propose a "mass transfer engineering" stepwise pre-oxidation strategy [104]. Through an "oxidation fragmentation re-oxidation" cyclic process, coupled with mechanical fragmentation to break through the dense surface oxidation shell, uniform oxidation of pitch particles from surface to core is achieved (Fig. 9a). This approach markedly enhances the oxygen content and crosslinking degree of pitch, suppresses graphitization, and yields HC materials with large interlayer spacings, abundant closed pores, low specific surface area, and high tap density. Zhang et al. have employed various pre-oxidation approaches to incorporate diverse oxygen-containing functional groups OFGs into coal molecules (Fig. 9c) [106]. In particular, the proposed alkali oxygen oxidation method not only dissolves organic components, averts the introduction of defects, and creates intra-coal pores, but also successfully introduces carboxyl functional groups into coal molecules. In a typical study, Hu et al. have used renewable tannin extract as the raw material [107]. The sulfuric acid treatment forms abundant and tunable closed nanopores in the carbon skeleton while introducing a large number of carbonyl groups. The prepared HC-1400 exhibits a reversible capacity of 360.96 mAh g^−1^ at 30 mA g^−1^ and maintains a discharge capacity of 169.2 mAh g^−1^ after 500 cycles at 1 A g^−1^. Additionally, Zhao et al. have treated sucrose-derived ordered mesoporous carbon with H_2_O_2_ to introduce carbonyl oxygen [108]. The OMC-75% electrode shows a charge capacity of 516 mAh g^−1^ at 0.05 A g^−1^. On the flip side, excessive oxidation may compromise the integrity of the carbon skeleton. Thus, gradient experiments on oxidation duration are required to determine the optimal modification window.
  In addition, pre-oxidation method can also be used to modify resin raw materials. Zhang et al. have used a simple pre-oxidation strategy to regulate the specific surface area and closed pore structure of phenol formaldehyde resin-derived HC anodes [105]. The key role of pre-oxidation is to promote precursor crosslinking and inhibit the rapid release of small molecules during carbonization. The slow release of pyrolysis gas can induce the formation of closed pores. The highly cross-linked structure not only reduces the specific surface area of HC but also inhibits the rearrangement of carbon atoms, increasing the disorder degree of HC. More importantly, pre-oxidation crosslinking can effectively reduce the pore size of cross-linked precursors, promote pore closure, and form HC materials with high closed pore volume. Compared with the control sample, its plateau capacity increased by approximately 90.9 mAh g^−1^.
  Pre-oxidation directly determines the necessity and effectiveness of post-treatment modifications. The controlled surface oxygen functional groups introduced via pre-oxidation facilitate the formation of a uniform, stable SEI during initial cycling, reducing the need for post-treatment surface coating and artificial SEI engineering to enhance ICE. The target carbonization technology and post-treatment performance goals impose reverse design constraints on the pre-oxidation protocol. For conventional tubular furnace carbonization, a moderate pre-oxidation degree is preferred to balance crosslinking efficiency and defect formation, whereas for flash Joule heating, minimal pre-oxidation is required to avoid excessive oxygen content that compromises precursor electrical conductivity.

### Component Regulation

Precursor composition regulation refers to the strategy of tailoring the chemical composition, structure of precursors, or incorporating specific additives prior to the fabrication of HC materials. This approach facilitates precise tailoring of the key microstructural characteristics of HC, including interlayer spacing, defect concentration, and porous architectures throughout the carbonization process, consequently providing additional active sites and diffusion pathways for the insertion and extraction of Na^+^ [109].

#### Impurity Treatment

Inorganic impurities in the precursor, such as oxides, salts, and silicates of elements like K, Ca, and Si, serve as pivotal byproducts that are challenging to eliminate during the pyrolysis and carbonization of HC [110]. Their chemical composition and spatial distribution characteristics exert a pronounced influence on the sodium storage performance of HC materials.

**Fig. 10:**
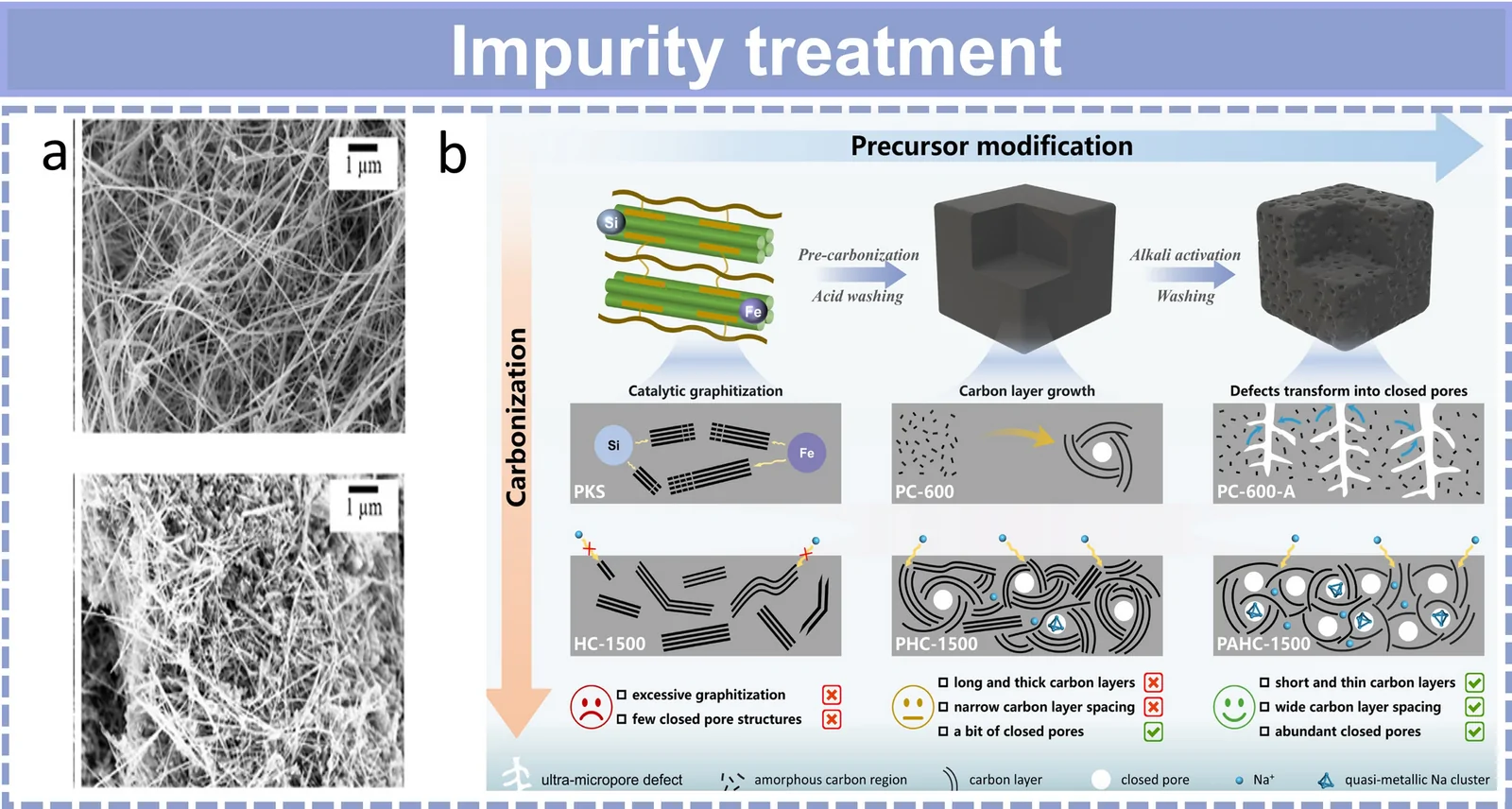
Influence of impurities on HCs. **a** Influence of impurity silicon on the structure [111]. Copyright 2022, Elsevier. **b** Influence of element regulation on HC pores [114]. Copyright 2025, Elsevier

Dupont et al. have demonstrated that Ca and Si elements exert a catalytic graphitization effect, which induces the formation of localized graphitic domains in HC [111]. However, the turbostratic structure and carbon purity of such samples are constrained, with SiC whiskers observable on the surface, ultimately leading to inferior reversible capacity (Fig. 10a). Sun et al. reported that most volatile components can be effectively eliminated during pyrolysis, thereby facilitating carbon conversion, while nonvolatile components are closely associated with carbon yield [112]. Zhao et al. have ground bituminous coal (RC) and eliminated minerals using hydrofluoric acid and hydrochloric acid, affording ultralow ash coal (BC) [113]. Subsequent processing of BC yields HC materials rich in ultra-micropores, which significantly enhances the plateau Na^+^ storage capability. Microbial technology has also been applied in impurity removal processes. Ren et al. have employed palm kernel shell as the precursor and enhanced the structural stability of the carbon matrix through pre-carbonization and acid washing treatments (Fig. 10b) [114]. This approach not only prevents excessive graphitization induced by internal impurities but also facilitates the formation of short and thin carbon layers, thereby remarkably improving the low potential capacity of HC. Li et al. have avoided conventional acid or alkali treatments for ash content reduction. Instead, they utilize Coriolus versicolor (CV) for microbial erosion preprocessing of bamboo [115]. CV mycelia grow within the bamboo matrix, reducing ash content while corroding bamboo fibers.

#### Extract Components

The properties of HC anode materials are dictated by the chemical composition and structure of their precursors [116]. However, natural biomass raw materials are often plagued by compositional complexity and structural inhomogeneity, thus inducing electrochemical performance variability in the derived HC materials, which cannot satisfy the requirements for batch uniformity and high performance of anode materials in practical SIBs [117]. Precursor modification enables the optimization of their composition and structure, laying a solid foundation for subsequent carbonization and performance enhancement.

**Fig. 11:**
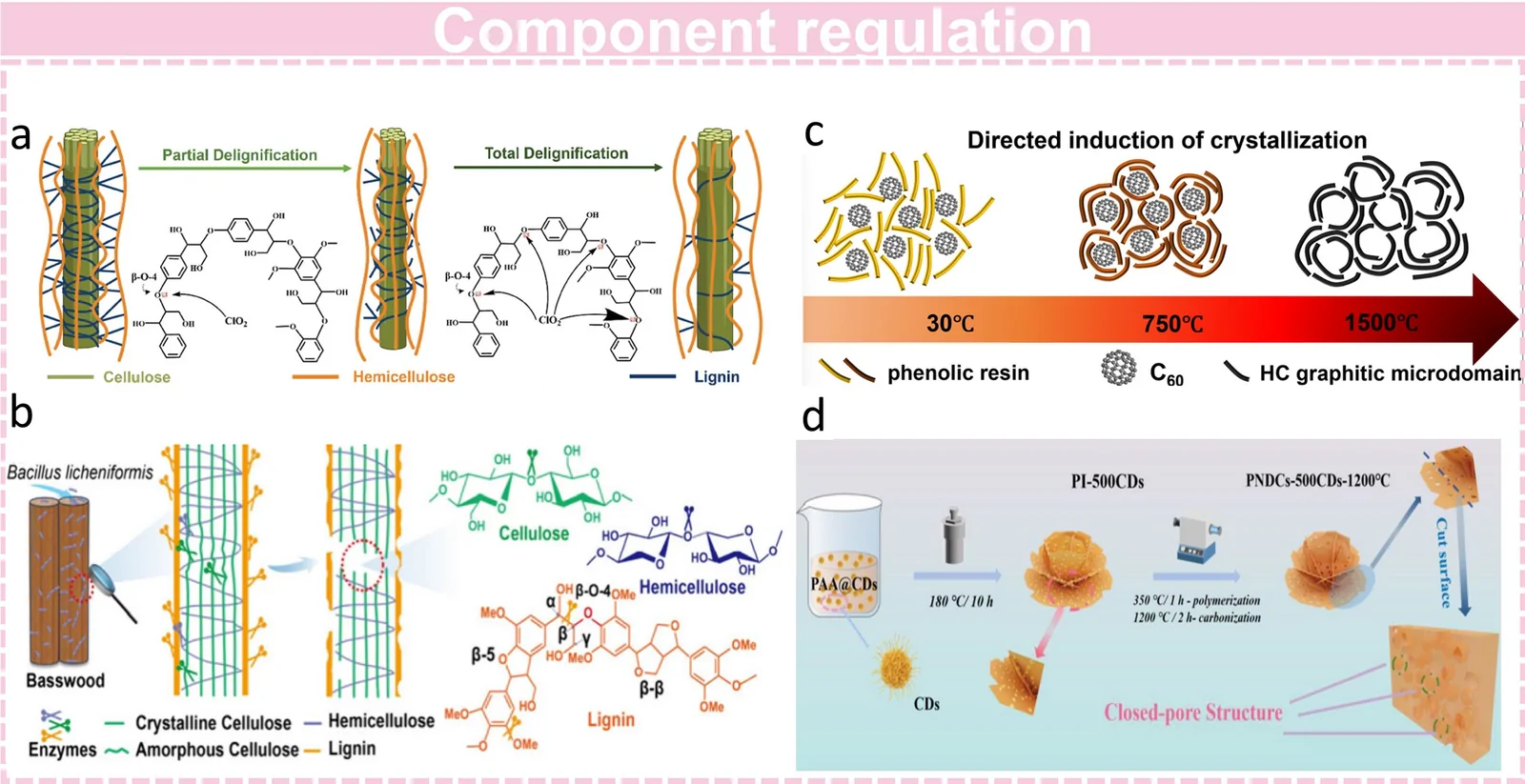
**a** Influence of exposed free radicals from delignification on sodium storage performance [119]. Copyright 2024, Wiley. **b** Microbial strategies for lignocellulose decomposition [124]. Copyright 2025, American Chemical Society. **c** Preparation of HC by mixing C60 with resin [130]. Copyright 2025, American Chemical Society. **d** Introducing carbon dots strategy [131]. Copyright 2023, Wiley

Building on this premise, cellulose, hemicellulose, and lignin are regulated and removed through various methods [118]. Wang et al. have achieved targeted delignification of natural bamboo using an acidic sodium chlorite solution (Fig. 11a) [119]. This delignification process exposes free radicals in the natural bamboo and enhances reactivity during carbonization. Sufficient free radicals facilitate the utilization of precursor fragments in the carbonization reaction, thereby forming well-developed carbon layers and abundant closed pores/ultra-micropores. Benefiting from the ultrahigh closed pore volume and hierarchical ultra-microporous structure, the optimized HC exhibits a high reversible capacity of 350 mAh g^−1^ at a current density of 20 mA g^−1^. Zhou et al. also have subjected raw waste wood-derived biomass to treatment with NaClO_2_ and NaOH to realize the stepwise elimination of hemicellulose and lignin, enhance crystallinity, and facilitate the formation of closed pores at relatively low temperatures [120]. Compared with untreated pyrolyzed carbon, the treated pyrolyzed carbon features more abundant closed pores, whose pore walls are composed of only 4 layers of graphitic carbon sheets. Meanwhile, Lan et al. pioneer the introduction of a deep eutectic solvent (DES)-assisted shearing strategy for the precise tailoring of a natural bamboo structure [121]. By simultaneously shearing and dissolving the amorphous components, DES regulates the crystallinity, order degree, and chain length of cellulose, which effectively modulates the pore structure of the derived HC. In another of their studies, Zhou et al. have developed a sulfuric acid hydrolysis approach for phloem cellulose from waste thermos bottle stoppers [122]. This method partially disrupts the long-chain structure of cellulose, reduces the graphitization degree of the derived carbon, and increases the closed pore content of the derived carbon. Additionally, Xu et al. have developed a synergistic strategy combining microwave assistance and acid treatment to achieve targeted regulation of the content of each component in natural lotus stems, modifying the spatial structure of the resulting HC [123]. Microwave assistance accelerates the reaction process and enables efficient decomposition of hemicellulose and lignin. XRD reveals that the cellulose crystallinity of the treated samples is significantly enhanced, attributable to the effective removal of amorphous components and impurities. Biological regulation has also been employed for the component modulation of precursors. Jiang et al. have utilized bio-enzymes secreted by lignocellulose-degrading bacteria to regulate the composition of lignocellulosic biomass (LCB) precursors at the molecular level, thereby enabling the precise tailoring of the microstructure of LCB-derived HC (LCB-HC) (Fig. 11b) [124]. This mild yet efficient enzymatic hydrolysis approach specifically achieves the partial depolymerization of bio-polymers in basswood, constructing graphitic domains with small curvature, abundant closed pores, and enlarged interlayer spacing. Such a structural configuration not only favors Na⁺ storage in the low-voltage plateau region but also accelerates ion transport kinetics, contributing to enhanced electrochemical performance.

#### Composite Components

Composite fabrication using different types of precursor materials integrates the advantages of individual components, enabling the preparation of HC materials with distinct microstructures and superior electrochemical performance. The composite of soft and HC raw materials, as well as the composite of nanomaterials such as carbon dots in HC raw materials, are two commonly used ways to regulate the structure of HC [125–127].

1. Composite of soft and HC raw materials
  Yin et al. have utilized phenol formaldehyde resin and pitch as precursors to tune the mass ratio of pitch (10–30%) and pyrolysis temperature (800–1300 °C) and regulate the micropore morphology and nanostructure of HC [126]. The mutual crosslinking between pitch and resin facilitates the formation of abundant short graphitic layers and reduces the specific surface area (SSA) and micropore content, thereby enhancing the sodium storage performance of the material. Another group of researchers has blended pitch with biomass hickory shells to fabricate soft carbon/ HC composites. The soft carbon component significantly reduces the SSA by sealing most open pores, and these sealed pores provide considerable low-voltage capacity for sodium storage. The optimized PSHC/SC-2 electrode exhibits a 68.4% higher specific capacity at a low current density of 50 mA g^−1^. Sun et al. have mixed pre-oxidized phenolic resin with pitch and subsequently calcined the mixture at 1200 ℃ for 1h under an argon atmosphere, yielding HC [128]. TEM divulges that the graphite domains of the mixed samples were more disordered, and the optimal sample PCHC 10 possesses abundant and appropriately sized closed pore structures. Zhao et al. have fabricated a weakly solvating interface by constructing a few-layer carbon coating enriched with sp^2^-hybridized carbon on HC via asphalt waste-derived precursors [129]. This tailored interface exhibits a substantially lower adsorption energy toward electrolyte solvent molecules compared to the pristine HC.

(2) Composite carbon nanomaterials
  Li et al. have mixed C60 molecules (with a diameter of 0.7 nm) with phenol formaldehyde resin, innovatively employing C60 to modulate the formation of closed pores in phenol formaldehyde resin-based HC (Fig. 11c) [130]. In another study, Huang et al. have in situ incorporated carbon dots CDs during the hydrothermal synthesis of polyimide PI, followed by high-temperature pyrolysis to produce HC materials (Fig. 11d) [131]. Clearly observed by TEM, the HC with CDs incorporated has abundant and uniform closed pores as well as a large interlayer spacing. This indicates that carbon dots can serve as a pore-forming additive to generate closed pore structures, thus suggesting a novel strategy to fabricate carbon materials with closed pore architectures. However, formidable interfacial compatibility challenges persist between composite components. Inadequate interfacial design can instigate persistent parasitic reactions, fostering the formation of unstable solid electrolyte interphase (SEI) films that severely compromise ionic transport kinetics and long-term cycling stability.
  The composition of raw materials will have a significant impact on the selection of subsequent pyrolysis processes. Precursors with high lignin content exhibit high crosslinking potential and thermal stability, enabling higher terminal carbonization temperatures to tune microcrystalline ordering, while cellulose-rich precursors require staged low-temperature pyrolysis to avoid excessive volatile release and pore structure degradation. For resin-pitch composite precursors, the mass ratio of resin to pitch directly defines the optimal carbonization heating rate, with higher pitch content requiring slower low-temperature heating to avoid phase separation and structural inhomogeneity. Meanwhile, component regulation directly governs the effectiveness and necessity of downstream post-treatment modifications. Precursors with tailored heteroatom content via component blending enable in situ bulk doping during carbonization, eliminating the need for post-treatment heteroatom doping to modulate electronic conductivity and sodium adsorption energy. The introduction of soft carbon precursors such as pitch can seal open pores during carbonization, reducing the need for post-treatment CVD coating to mitigate irreversible interfacial side reactions and enhance ICE.

#### Pre-doping

Pre-doping represents a strategic intervention at the precursor stage, analogous to a controlled genetic modification process, wherein specific heteroatoms are incorporated during the initial formation of the carbon skeleton. Its core merit lies in avoiding the physical damage to the carbon skeleton caused by post-treatment doping; by leveraging the chemical interactions between heteroatoms and the precursor, it simultaneously constructs targeted defect structures, optimizes electronic state distribution, and interfacial properties during carbonization [58, 132]. This provides sufficient active sites for sodium storage, accelerates ion and electron transport kinetics, and improves electrode-electrolyte interfacial compatibility, thereby synergistically enhancing the reversible capacity, ICE, and cycling stability of HC.

**Fig. 12:**
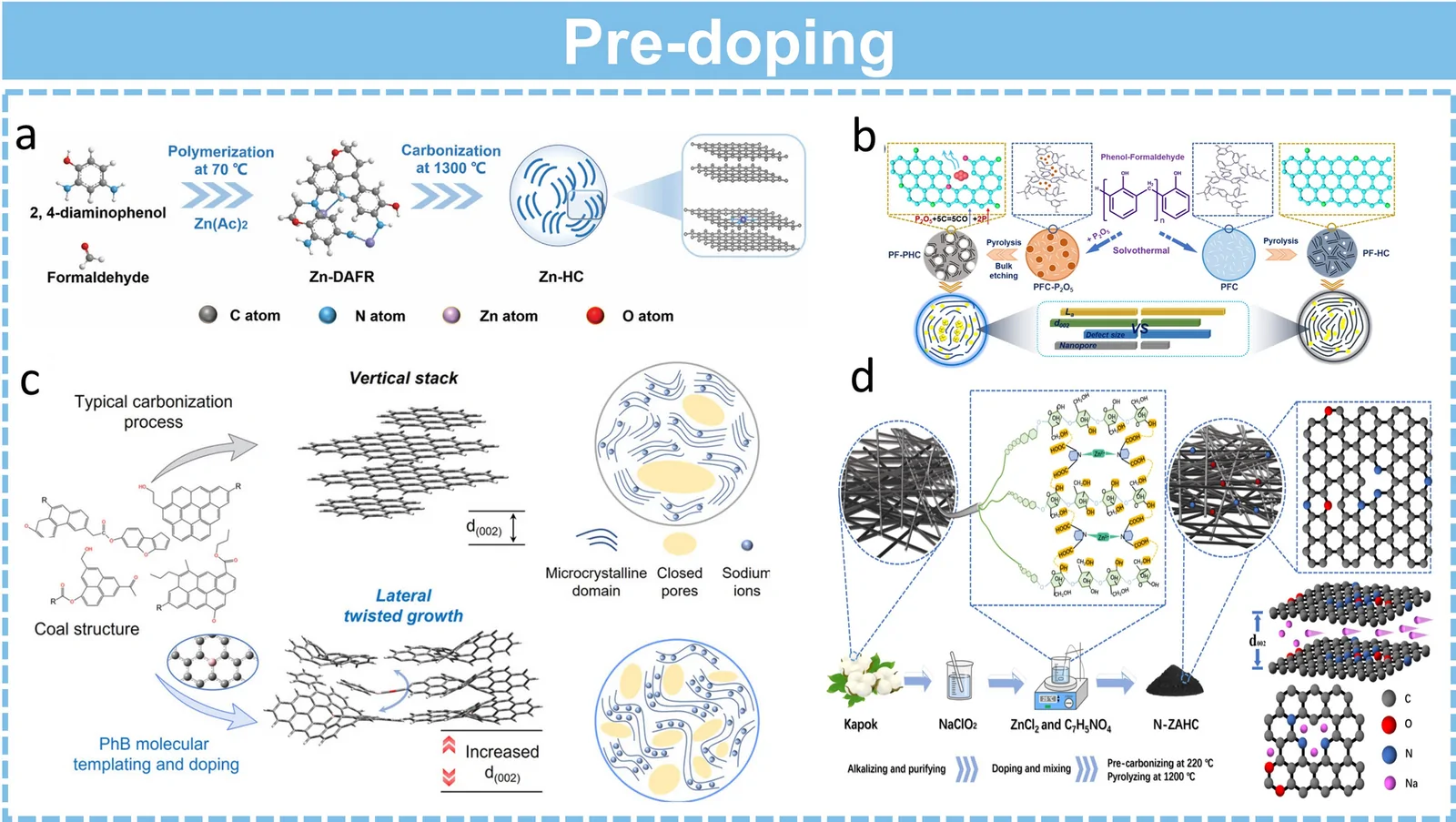
**a** Zinc single-atom-regulated HC [133]. Copyright 2023, Wiley. **b** Phosphorus doping strategy [135]. Copyright 2024, Wiley. **c** Boron doping strategy [136]. Copyright 2025, Wiley. **d** Nitrogen doping strategy [137]. Copyright 2024, Elsevier

For instance, Lu et al. have reported a zinc single atom doped HC material Zn HC [133]. The doping of zinc single atoms enables the modulation of the bulk and surface structures of HC (Fig. 12a). The optimized Zn-HC exhibits a larger carbon interlayer spacing (d_002_ = 0.408 nm), well-suited nanopores (diameter ~ 0.8 nm), and lower defect content, facilitating rapid sodium ion intercalation and pore filling. In another work, Qiu et al. develop nickel single atom modified nitrogen and phosphorus co-doped HC Ni-NPC with a nickel content of approximately 5.65 wt% [134]. Through theoretical calculations and kinetic analyses, they demonstrate that nickel single atoms serve as promoters for Na^+^ diffusion. Liu et al. have utilized phenolic resin as the carbon precursor and phosphorus pentoxide P_2_O_5_ as a bifunctional sacrificial template and doping source (Fig. 12b) [135]. During pyrolysis, P_2_O_5_ acts as an endogenous gas phase template to induce pore mouth shrinkage and generate a closed pore network. A small amount of residual phosphorus atoms is incorporated into the carbon skeleton to achieve defect passivation, accompanied by an increase in interlayer spacing. In a separate study, Zhang et al. have employed phenylboronic acid PhB as a molecular template and doping element to regulate the microcrystalline structure of coal-based HC (Fig. 12c) [136]. The *π*–*π* interactions between phenylboronic acid and coal aromatics suppress excessive stacking, while boron B doping perturbs charge distribution and introduces intradomain defects. These effects lead to the formation of HC materials with twisted turbostratic domains featuring expanded interlayer spacing. Furthermore, Zhang et al. have subjected kapok to alkaline treatment to open cellulose molecular chains, mixed 2,6-pyridinedicarboxylic acid (as the nitrogen source) with cellulose macromolecules to prepare kapok-based HC with efficient nitrogen doping, and optimized the formation of C=O bonds in the carbon network (Fig. 12d) [137].

Nevertheless, while pretreatment doping demonstrates significant value in optimizing the sodium storage performance of HC, it requires a high degree of compatibility with precursor characteristics and carbonization processes; otherwise, negative coupling effects are likely to occur. Precision control of the concentration, chemical state, and spatial distribution of heteroatoms remains challenging. In practice, dopant agglomeration and incomplete reaction with the precursor often result in local enrichment or inhomogeneous distribution of heteroatoms. Local enrichment may induce excessive carbon skeleton defects, exacerbating electrolyte side reactions; inhomogeneous distribution, on the other hand, can cause fragmentation of sodium storage active sites, reducing capacity stability [112, 138, 139]. Presently, a general theoretical framework remains elusive for quantitatively correlating specific heteroatoms with critical structural parameters, including closed‑pore development, interlayer‑spacing expansion, and sodium‑storage mechanisms. Consequently, future research should shift its focus from simply identifying which heteroatoms are introduced to elucidating the dynamic roles these atoms assume during the carbonization process.

Compared with the post-doping method, the pre-doping strategy introduces heteroatoms into the precursor before carbonization, which can fundamentally avoid structural damage caused by secondary processing and achieve a more uniform doping distribution at the atomic scale. Non-metallic dopants (such as nitrogen and phosphorus) mainly exert their effects by regulating local electron density and generating pseudocapacitive active sites. And metal single atom dopants (such as Zn and Ni) can act as catalytic centers to change the carbonization pathway and promote the formation of specific pores or defects. The challenge of pre-doping lies in achieving precise and reproducible control of the doping process. The thermal stability and chemical reactivity of the dopant must be carefully matched with the carbonization conditions; otherwise, agglomeration, volatilization, or the formation of inert phases may occur.

Pre-doping has a significant impact on the carbonization process itself. Metal heteroatoms with catalytic graphitization can reduce the activation energy of carbon atom rearrangement, thereby forming ordered graphite microdomains at lower carbonization temperatures. Non-metallic heteroatoms inhibit the stacking of graphite layers, requiring higher final temperatures to achieve moderate microcrystalline order. Post-doping occurs after carbonization is complete and cannot interfere with the formation process of the HC skeleton.

More importantly, pre-doping directly determines the demand and effectiveness of subsequent post-treatment modifications. The pre-doping of the bulk phase optimizes the intrinsic electronic conductivity and sodium ion adsorption energy of the HC matrix from the source, fundamentally alleviating the limitation of rate performance and reducing the necessity of surface doping for post-treatment to improve charge transfer kinetics; Meanwhile, pre-doping can form a uniform defect distribution within the bulk phase during the carbonization process, minimizing the need for post-treatment surface passivation to repair local irreversible defects. Ultimately, pre-doping regulates the electronic structure of the bulk phase, while post-doping optimizes the surface interface properties.

From a strategic selection perspective, pre-doping is preferred when the primary optimization goal is to enhance the intrinsic rate capability and structural stability of HC, especially for precursors with low intrinsic reactivity and poor conductivity. Post-doping is strategically advantageous when the core goal is to improve ICE and interfacial stability, particularly for HC materials with already optimized bulk pore and graphitic structures, where post-doping can achieve interfacial modification without disrupting the pre-established bulk performance. For comprehensive performance optimization, a cross-stage synergistic doping strategy combining pre-doping and post-doping is highly recommended: Pre-doping is used to regulate the bulk electronic structure and ion diffusion kinetics, while post-doping is employed to passivate surface defects and optimize the interfacial properties, achieving synergistic improvement of all core performance metrics across the full preparation chain.

### Pore-forming Treatment

A pore-forming treatment refers to the process of introducing pore structures into precursors via physical or chemical approaches during the fabrication of HC anode materials, aiming to optimize the sodium storage characteristics of HC. The primary objective is to construct HC materials with hierarchical pore channels (micropores < 2 nm, mesopores 2–50 nm, macropores > 50 nm), optimized specific surface area, porosity, and ion transport pathways [140–143]. Primary pore structures generated via pore-forming processes, including activation and template assisted synthesis, commonly suffer from broad pore size distribution, excessive open porosity, and poorly ordered pore wall microstructure, which exacerbate irreversible interfacial side reactions and compromise the comprehensive electrochemical performance of the final material. Accordingly, two differentiated post- pore-forming regulatory pathways have been developed to address these limitations, namely pore narrowing strategy and pore filling strategy. The pore narrowing strategy delivers dimensional precision regulation and framework reinforcement of the existing pore structure via in situ optimization, which retains the intrinsic interconnection of the pore network. The pore filling strategy achieves directional modification of pore accessibility and open-closed attribute via interface engineering, which focuses on the precise control of the contact interface between the pore structure and electrolyte. The detailed regulatory mechanisms and process correlations of pore filling strategy are systematically elaborated in the subsequent pore filling section of this review.

#### Activation

Activation-assisted pore creation is a technique that employs gases (e.g., CO₂, water vapor) or chemical reagents (e.g., KOH, ethanol) for selective etching of carbon skeletons at elevated temperatures, thereby incorporating microporous or mesoporous architectures into HC [144].

**Fig. 13:**
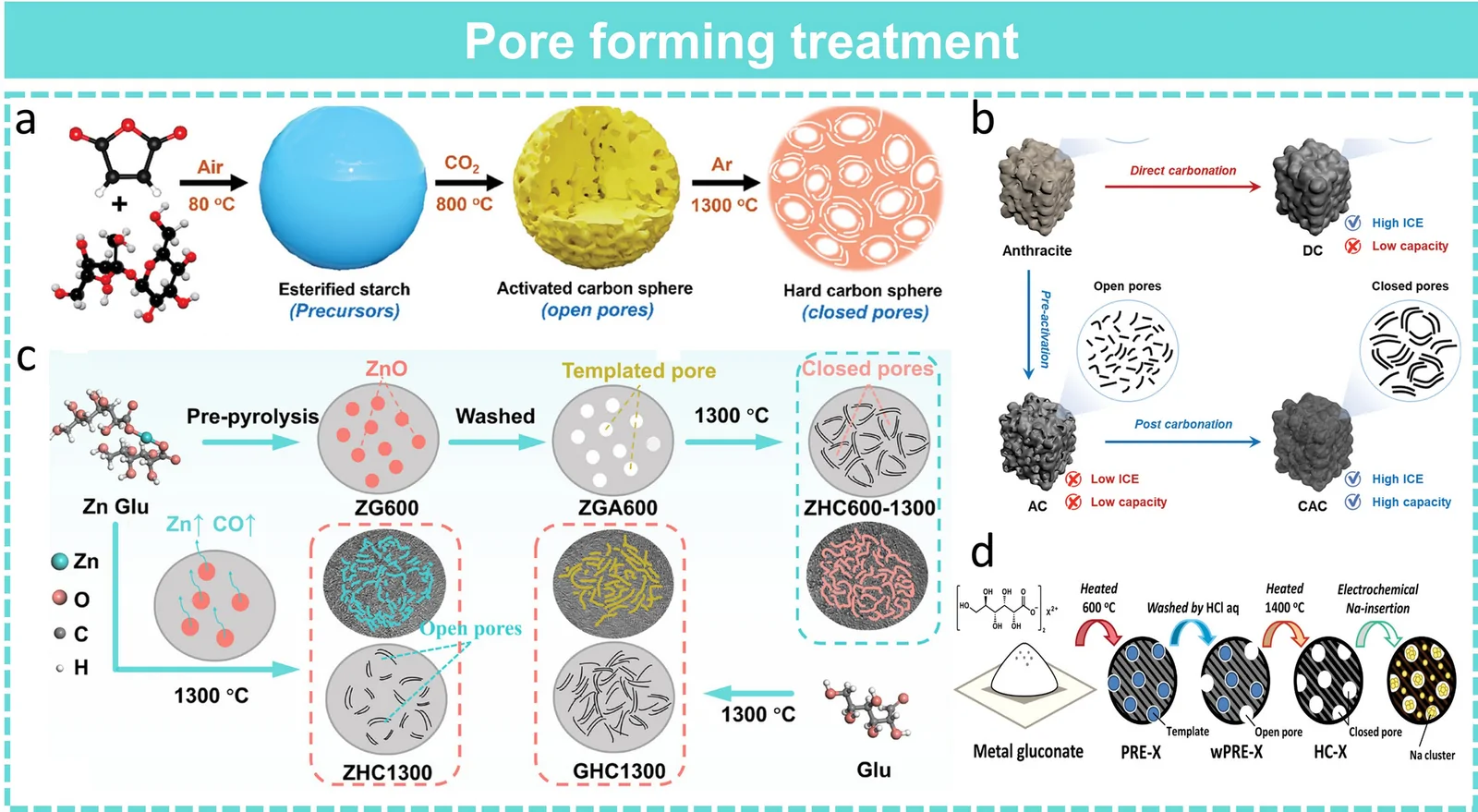
**a** CO_2_-etching pore formation [145]. Copyright 2023, Wiley. **b** Alkali activation pore formation [146]. Copyright 2022, Wiley. **c** ZnO template pore formation [147]. Copyright 2025, Wiley. **d** Different template-assisted pore formation [149]. Copyright 2023, Wiley

Remarkably, Zheng et al. have utilized esterified starch for CO₂ etching activation at a relatively low temperature (800 °C), generating abundant open pores (Fig. 13a) [145]. This is followed by carbonization at an elevated temperature (~ 1300 °C) with the atmosphere switched from CO_2_ to Ar, where the open pores are converted into closed pores, yielding HCMP–CO_2_ with a high density of closed pores. The numerous closed pores endow it with an ultrahigh specific capacity of 487.6 mAh g^−1^. The corresponding plateau capacity (351 mAh g^−1^) is substantially higher than that of the unetched starch-derived HC (193 mAh g^−1^). Wang et al. have adopted KOH-aided chemical activation, which incorporates numerous open nanopores and disordered carbon configurations into the resulting carbon product (Fig. 13b) [146]. This is followed by further high-temperature carbonization to tune the microcrystalline and pore characteristics of the final carbon product. In the course of high-temperature carbonization, the predefined open porous architectures effectively inhibit the long-range ordered growth of carbon crystallites, thereby promoting the generation of abundant closed pores and contributing to Na^+^ storage in the low-voltage plateau region.

#### Template Method

Ren et al. have employed homologous zinc gluconate in conjunction with a pre-carbonization strategy to engineer an HC model, featuring open pores, sub-0.7 nm ultra-micropores, and closed pores (Fig. 13c) [147]. This approach precisely tunes the concentrations of ultra-micropores and closed pores while suppressing the formation of open pores. Qiu et al. have utilized zinc gluconate ZG as the precursor, achieving the generation of open pores and their conversion to closed pores via direct high-temperature pyrolysis [148]. They further elucidate that the process of closed pore formation from ZG during pyrolysis comprises four stages: zinc oxide embedding, precipitation growth, outgassing, and open pore closure. Leveraging the pore structure of templates directs the formation of HC materials, and subsequent template removal post-carbonization yields HC with tailored pore architectures. Igarashi et al. have systematically investigated the utilization of MgO, ZnO, and CaCO_3_ as nanopore templates for HC modulation, fabricating HC materials denoted as HC-Mg, HC-Zn, and HC-Ca (Fig. 13d) [149]. The ZnO template is particularly effective. Small-angle X-ray scattering (SAXS) reveals that the average pore sizes of HC-Mg and HC-Zn are 1.25 and 1.44 nm, respectively. In contrast, HC-Ca exhibits a shoulder peak with extremely low intensity in its SAXS pattern. Guo et al. have employed collagen fibers as the precursor and in situ generated Mg(OH)_2_ as the template to modulate the derived carbon material (CMK700-3) [150]. During the pyrolysis stage, collagen undergoes gradual carbonization at moderately elevated temperatures and adheres to the template surface, thus forming a porous carbon material with a rich pore network, with pore size dependent on template dimensions. BET and electrochemical analyses reveal that Mg(OH)_2_ particles promote the formation of mesopores and enhanced Na^+^ diffusion kinetics.

In recapitulation, pore-forming treatment undoubtedly promotes the formation of dense pore structures and can provide abundant active sites for Na^+^ storage and optimizes effective Na^+^ transport kinetics to enhance sodium storage performance. However, additional coating procedures are necessitated to reduce the specific surface area of these porous carbon. Hierarchical pore frameworks with interconnected mesopores facilitate uniform infiltration of liquid-phase doping reagents and homogeneous gas-phase deposition during CVD post-treatment, enabling conformal pore wall coating without pore blockage. Conversely, excessive microporosity generated via uncontrolled activation leads to uneven deposition and pore blockage during post-treatment CVD, compromising ion transport kinetics. The rational design of closed pore frameworks via alkaline activation combined with pore filling (CVD) maximizes both plateau capacity and ICE. This tight relationship confirms that pore-forming treatment must be designed in concert with downstream pyrolysis and post-treatment processes, rather than optimized as an isolated structural modification step.

## Mid-pyrolysis Processes

In the fabrication of HC materials, carbonization processes encompass the systematic regulation of processing parameters such as carbonization temperature, heating rate, holding time, cooling rate, as well as carbonization atmosphere. These key parameters are interconnected and act synergistically, collectively governing key characteristics of HC materials such as the degree of crystallinity, graphitization, defect configuration, carbon interlayer spacing, specific surface area, and porous architecture [151–153].

### Conventional Slow Heating Carbonization

**Fig. 14:**
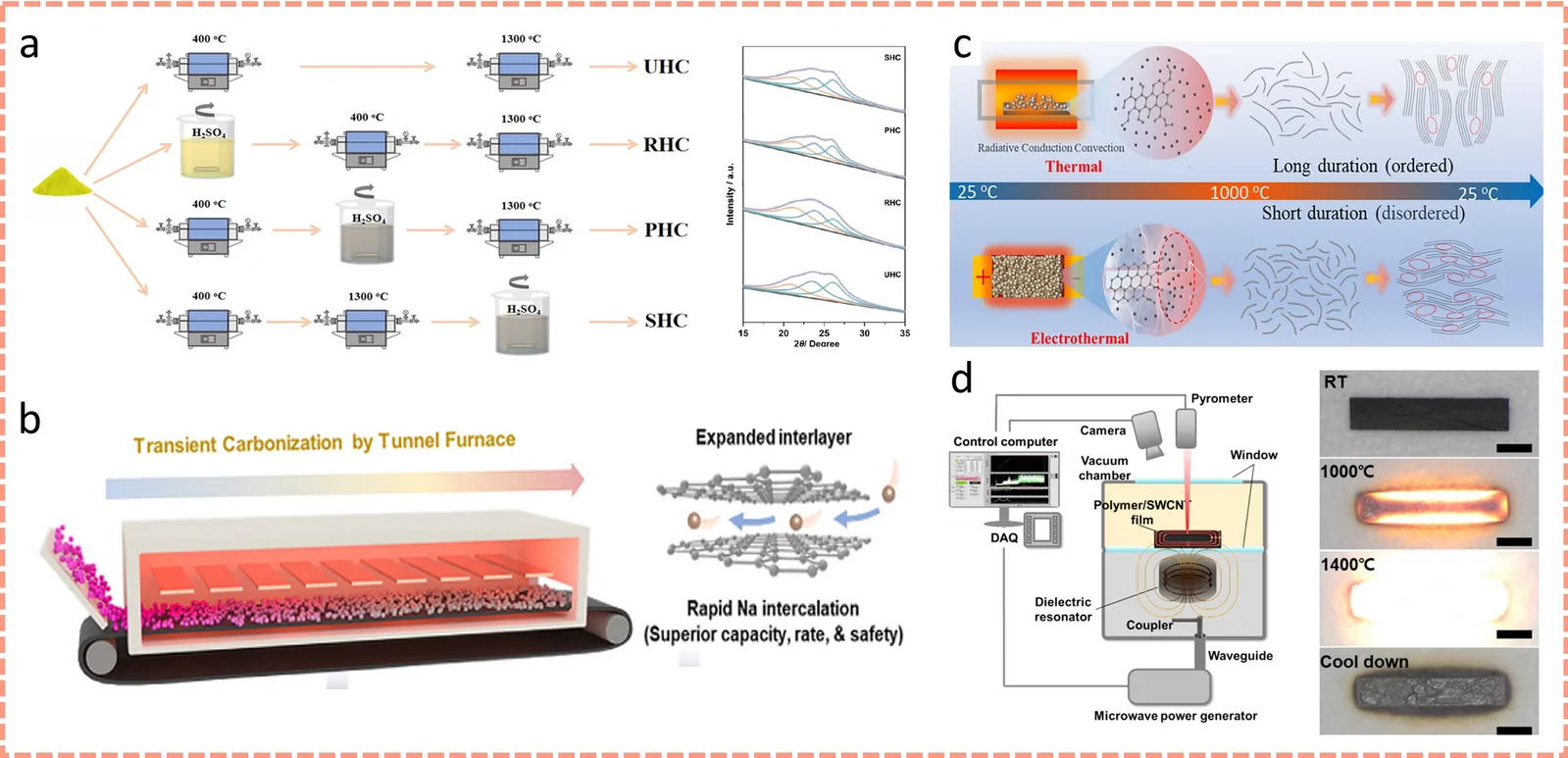
**a, b** Traditional tube furnace pyrolysis process [154]. **c** Joule heating pyrolysis process [33]. Copyright 2025, Elsevier. **d** Pre-carbonization treatment coupled with joule heating [164]. Copyright 2025, Wiley. **e** Thermodynamic analysis of joule heating process [165]. Copyright 2025, Wiley. **f–g** Microwave-assisted Joule heating [166]. Copyright 2024, Elsevier

Conventional slow heating carbonization stands as the most prevalent method for HC synthesis, typically employing thermal processing equipment such as tube furnaces and box furnaces in the laboratory. It is defined by relatively low heating rates with heating rates in the range of several to tens of degrees Celsius per minute, as well as prolonged durations, typically lasting from hours to tens of hours. During this slow heating and prolonged soaking process, organic moieties in the precursor undergo sequential decomposition and polycondensation, enabling the stepwise conversion into amorphous HC. Wu et al. have prepared kilogram-scale industrial-grade HC by performing acid washing on the precursor at different stages, followed by high-temperature carbonization in a tube furnace (Fig. 14a, b) [154]. In addition, Sun et al. have utilized thermosetting phenol formaldehyde resin to modulate sufficiently large interlayer spacing of carbon layers and the formation of closed micropores (~ 2.1 nm) under high pyrolysis temperatures [155]. TEM reveals that as the carbonization temperature gradually increases from 900 to 1500 °C, the microstructure of HC exhibits an evolutionary trend from disordered carbon layers to graphitoid domain alignment. Meanwhile, short graphitoid layers morph into long-range layers, while long-range curved graphitoid domains induce the formation of numerous visible closed nanopores. Prykhodska et al. have synthesized HC samples featuring the largest specific surface area, the highest defect density, and a highly asymmetric pore structure by precisely controlling the heating rate, thereby significantly enhancing the sodium storage performance [156].

Nevertheless, conventional carbonization exhibits inherent limitations. It offers limited capability for precision tuning of HC microstructures and confronts difficulties in realizing rapid and accurate temperature control, which are two key constraints for practical, scalable, and high-performance HC production.

### Pyrolysis Atmosphere

The pyrolysis atmosphere modulates surface chemistry and bulk structure via gas–solid reactions. Inert atmospheres such as argon or nitrogen minimize secondary reactions, preserving a relatively intact carbon framework with moderate graphitic ordering and low surface defect density, which balances ICE and cycling stability by suppressing excessive solid electrolyte interphase formation [26, 28, 36]. Oxidative atmospheres such as carbon dioxide induce controlled matrix etching, generating interconnected micro- and mesopores that shorten ionic migration pathways and enhance rate capability, though the enlarged specific surface area triggers irreversible side reactions that reduce initial efficiency. Reductive atmospheres such as hydrogen promote defect passivation and graphitic growth, improving electronic conductivity and structural rigidity, but the reduced defect density limits active sites for sodium adsorption, leading to a decrease in capacity. Lu et al. have proposed an In situ gas phase structural engineering technique, in which organic free radicals generated from the decomposition of ethyl acetate vapor interact with defect sites in walnut shell-derived HC, inducing distinct local ordering of nanodomains. The synergistic crosslinking effect simultaneously optimizes the structural order and nanodomain distribution of the carbon framework to facilitate the formation of a stable solid electrolyte interphase and enhance the reversibility of sodium storage [37]. Zheng et al. have coupled CO_2_ etching with carbonization to elicit abundant confined closed nanopores within the resultant HC, with the sole manipulation throughout the entire synthesis being the switch of gas atmosphere from carbon dioxide to argon, thus laying a feasible foundation toward facilitating the commercialization of high energy density SIBs [145].

Atmosphere switching acts as a feasible strategy for precise tuning of HC structure and properties. However, it exhibits some drawbacks that restrict its practical application and commercialization progress. The primary drawback lies in elevated process complexity and increased cost for scalable fabrication, precise control of switching timing, gas flow rate, and duration demands advanced equipment and strict operation protocols, adding a substantial burden to large-scale production and impeding commercialization progress. Secondly, maintaining structural homogeneity across the carbon matrix becomes challenging, as mismatched switching parameters easily induce uneven distribution of pores and graphitic domains, leading to localized performance divergence and degraded batch consistency. Moreover, complete synergistic optimization of all core electrochemical metrics remains elusive. Atmosphere switching mitigates partial performance tradeoffs but fails to reconcile all indicators. The enhanced rate capability from porous structure usually comes at the cost of ICE, while improved structural rigidity from inert atmosphere treatment may restrict reversible sodium storage capacity to a certain extent. In addition, slight deviations in the atmosphere switching operation will trigger irreversible structural defects, further impairing the repeatability and reliability of HC products in practical battery systems.

### Pyrolysis Rate

The pyrolysis rate governs the kinetic pathway of carbon atom rearrangement. Slow pyrolysis allows sufficient time for atomic ordering, fostering long-range graphitic microcrystalline with narrow interlayer spacing and low defect density to enhance mechanical rigidity and long-term cycling stability by mitigating volumetric expansion [157]. In contrast, rapid pyrolysis restricts atomic rearrangement, generating short-range disordered microcrystalline with expanded interlayer spacing and abundant closed pores. This configuration elevates reversible sodium storage capacity via additional active sites and confined deposition spaces but impairs electronic conductivity and ionic diffusion kinetics, resulting in inferior rate capability. Li et al. have regulated pyrolysis rates across distinct stages to modulate the defect density within the HC framework, thereby enhancing the capacity of both the plateau and slope regions while mitigating irreversible capacity loss [94]. They confirm that a slow heating rate at low temperature below 400 °C allows complete release of CO, CO_2,_ and CH_4_, thereby boosting abundant micropore formation in the carbonaceous intermediate. Yang et al. have performed a comparative study on slow pyrolysis at 5–20 °C min^−1^ and rapid pyrolysis at heating rates exceeding 100 °C min^−1^ [158]. Slow pyrolysis enables gradual devolatilization, yielding a carbon framework with low oxygen content and limited porosity. In contrast, rapid pyrolysis triggers explosive decomposition to facilitate ester group formation and suppress aromatic ring aggregation.

Overall, a slow pyrolysis rate does not exhibit absolute superiority during HC pyrolysis, while a rapid pyrolysis rate is not inherently inferior. The key lies in tailoring the heating rate to distinct temperature stages rather than adopting a uniform rate throughout the process. Different temperature stages require specific heating rate adaptation to optimize HC structural evolution and electrochemical performance.

### New Carbonization Process

To address the limitations of conventional carbonization, researchers have developed various advanced carbonization techniques to achieve more precise control over the structure and performance of HC. Flash Joule heating (FJH) emerges as an innovative carbonization technology that leverages the Joule heating principle: Through the instantaneous application of high current, precursors are rapidly heated to carbonization temperatures within an extremely short timeframe [159]. This technique enables carbonization in milliseconds, effectively mitigating issues such as the expansion of structural defects caused by prolonged high-temperature exposure in conventional carbonization processes.

Huang et al. have adopted relatively low temperature pyrolysis (700 °C for 1h) to remove impurities, followed by the application of joule heating (1950 °C for 22 s) to promote rapid ordered carbon crystallization while precluding significant graphitization, thus realizing the expansion of interlayer spacing and a reduction in process energy consumption by approximately 80% (Fig. 14c) [33]. The obtained expanded carbon EC exhibits a larger grain size and interlayer spacing, thus enabling higher specific capacity and excellent rate capability. Wang et al. have increased the yield of HC by utilizing Joule heating [160]. Luo et al. have utilized lignin as the carbon source and employed both conventional carbonization and rapid Joule heating techniques to successfully fabricate HC materials with distinct graphitization structures [161]. The J1600 sample exhibits the highest degree of graphitization and most ordered graphitic layers, correlating closely with the excellent electrochemical properties. Electrochemical analyses revealed that this electrode displays the lowest nucleation overpotential and the highest Coulombic efficiency during sodium deposition. Certain elements deemed detrimental in traditional processes may exhibit advantageous effects under joule heating conditions. Capitalizing on inherent potassium content within biomass precursors, Wu et al. have employed Joule heating technology to optimize the pore structure of HC and mitigate its graphitization degree [162]. Their study challenges the conventional understanding that potassium exerts an adverse effect on HC and for the first time demonstrates that under specific heating conditions, e.g., carbothermal shock method CTS, potassium can effectively function as a porogen and broaden the interlayer spacing, thus effectively improving the overall sodium storage capacity of HC at low potentials. Joule heating, however, entails high costs, and materials with poor conductivity may require pre-treatment to improve their electrical conductivity; otherwise, effective regulation cannot be achieved. To address this, some researchers combined preheating treatment with flash Joule heating (FJH) technology to rapidly generate abundant closed pore structures and expand the carbon interlayer spacing [163]. Qiu et al. have employed a two-step method combining preheating treatment and joule heating (Fig. 14d) [164]. Firstly, they transform brittle biomass into a framework with high carbonization stability; subsequently, the high temperature generated via millisecond-scale heating and cooling facilitates the rapid formation of closed pore structures in the material, which significantly enhances the sodium storage performance of HC. Closed pores provide abundant storage sites for sodium ions, while preheating treatment creates convenient channels for sodium ion transport by increasing the interlayer spacing. The sample subjected to both preheating and FJH treatment (HC600-J-1500) exhibits higher short-range order, characterized by the combination of local graphitized regions and amorphous carbon domains. This strategy not only significantly improves the yield of HC materials (up to 14 times) but also endows them with excellent reversible capacity (377 mAh g^−1^) and ICE (93.3%) in Na^+^ storage. Huang et al. have employed Joule heating using a heating rate of 100 °C s^−1^ to accomplish the rapid conversion from the amorphous state to graphite microcrystals in carbon materials in a brief timeframe, avoiding structural degradation issues prevalent in conventional high-temperature sintering, such as excessive phase transition from amorphous to graphitic phase and pore collapse (Fig. 14e) [165]. This thus preserves the structural integrity of the HC material, and the optimized HC synthesized at 1,000 °C has an outstanding sodium storage capacity. Ryoo et al. have proposed a novel method for the rapid preparation of HC anode materials via microwave-induced heating MIH [166], which is amenable to large-scale production. Employing circular heating plates and array resonators, it enables the continuous production of homogeneous HC anodes (Fig. 14f, g).

FJH represents a paradigm shift in carbonization, from a slow thermodynamic process to an ultrafast kinetic one. By minimizing time at high temperature, it uniquely suppresses the growth of graphitic domains while potentially preserving more defects and pores, leading to expanded interlayer spacing. This challenges the conventional wisdom that higher temperatures always lead to more ordered structures. However, its critical limitation is the requirement for precursor electrical conductivity, often necessitating pre-treatment (e.g., mixing with conductive agents).

Despite the remarkable laboratory-scale performance of FJH- HC, a critical assessment of its industrial scalability and economic viability reveals several non-negligible technical and engineering bottlenecks that have not been fully addressed in current research.

First, the core challenge for FJH scale-up is achieving uniform heating in large-batch production. Laboratory-scale FJH relies on the Joule heating of a small-volume carbon precursor compact between two electrodes, with extremely fast heating and cooling rates that are difficult to replicate in large-volume reactors. In scaled-up systems, uneven current distribution will lead to significant temperature gradients within the precursor bulk, resulting in heterogeneous graphitization, pore structure distribution, and severe batch-to-batch performance fluctuations, which is fatal for the mass production of battery-grade anode materials that require strict consistency. Second, the widely cited 80% energy saving of FJH compared to conventional tube furnaces is exclusively based on laboratory-scale small-batch reaction calculations. In industrial continuous production, this energy saving ratio will be significantly reduced. Laboratory-scale FJH systems ignore the energy consumption of auxiliary equipment, including vacuum systems, high-voltage power supplies, and cooling systems, which account for a large proportion of the total energy consumption in industrial continuous production. Moreover, most precursors with poor conductivity require pre-carbonization pretreatment to achieve effective Joule heating, which further offsets the energy saving of the FJH process itself. Third, the industrial application of FJH faces severe challenges in equipment cost, operational safety, and continuous production. The high-current, high-voltage pulsed power supply required for large-scale FJH has extremely high equipment investment and maintenance costs, and the harsh operating conditions pose significant safety risks in industrial workshops. Currently, there is no mature continuous FJH production line for HC anode materials, and the transition from batch laboratory-scale synthesis to continuous industrial production remains a formidable technical barrier.

In summary, FJH represents a promising innovative carbonization technology for the rapid synthesis of HC with unique microstructures, but its industrial application requires breakthroughs in large-batch uniform heating, low-cost continuous production equipment, and process stability. Future research on FJH should shift from laboratory-scale performance demonstration to critical engineering feasibility assessment, to truly bridge the gap between academic research and industrial application.

As the core stage for forming HC’s intrinsic sodium storage framework, the entire mid-pyrolysis process system, including conventional carbonization, atmosphere regulation, heating rate design, and novel ultrafast carbonization technologies such as flash Joule heating, exhibits inherent bidirectional coupling with full upstream pretreatment protocols and downstream post-treatment modification strategies. Its process parameters are fundamentally dictated by precursor physicochemical properties modulated via upstream pretreatment, while simultaneously defining the implementation boundary, necessity, and modification efficacy of subsequent downstream post-treatment processes. Precursor crosslinking density, thermal stability, and pore structure tuned via upstream pre-oxidation, chemical crosslinking, pre-carbonization, and pore-forming treatment directly govern the optimal heating rate, terminal temperature, holding time, and atmosphere control strategy during pyrolysis. Pre-carbonization reduces precursor volatile content to enhance batch consistency in industrial continuous carbonization, while flash Joule heating relies on pre-carbonization pretreatment to ensure uniform heating and avoid structural heterogeneity. Meanwhile, the microcrystalline ordering, defect density, and pore topology formed during carbonization directly determine downstream post-treatment strategy selection and implementation efficacy. Downstream post-treatment performance objectives also impose reverse design constraints on carbonization process design. A representative example is unilaterally expanding interlayer spacing without consideration of precursor adaptability or post-treatment feasibility. This confirms that carbonization must be designed as the core coordinating step in the holistic full chain framework, rather than a standalone structural formation process.

## Post-treatment

HC post-treatment centers on the precision modulation of surface chemical properties of the carbon matrix and interfacial structures. By eliminating surface defect states and constructing efficient ion/electron transport networks, these techniques achieve kinetic optimization of the electrode-electrolyte interface and suppression of side reactions. Based on differences in their action mechanisms and implementation pathways, post-treatment technologies can be systematically categorized into five major types, including surface functional group regulation, post-doping, pore-filling, surface coating, and pre-sodiation. Each strategy synergistically enhances the electrochemical performance of HC anodes through distinct physicochemical modification mechanisms.

### Surface Functional Group Regulation

Surface functional group regulation aims to tailor the surface chemical properties of HC materials, thereby enhancing their compatibility with electrolytes and electrochemical interfacial stability. This strategy facilitates the formation of a robust SEI film, mitigates side reactions, consequently improving the cycling stability and rate capability of sodium ion batteries. Of particular significance, surface functional groups such as hydroxyl, carboxyl, and carbonyl groups exert a profound influence on the electrochemical performance of HC anodes [157]. These groups not only modulate the surface properties of HC but also tailor its electronic structure and ion transport characteristics.

**Fig. 15:**
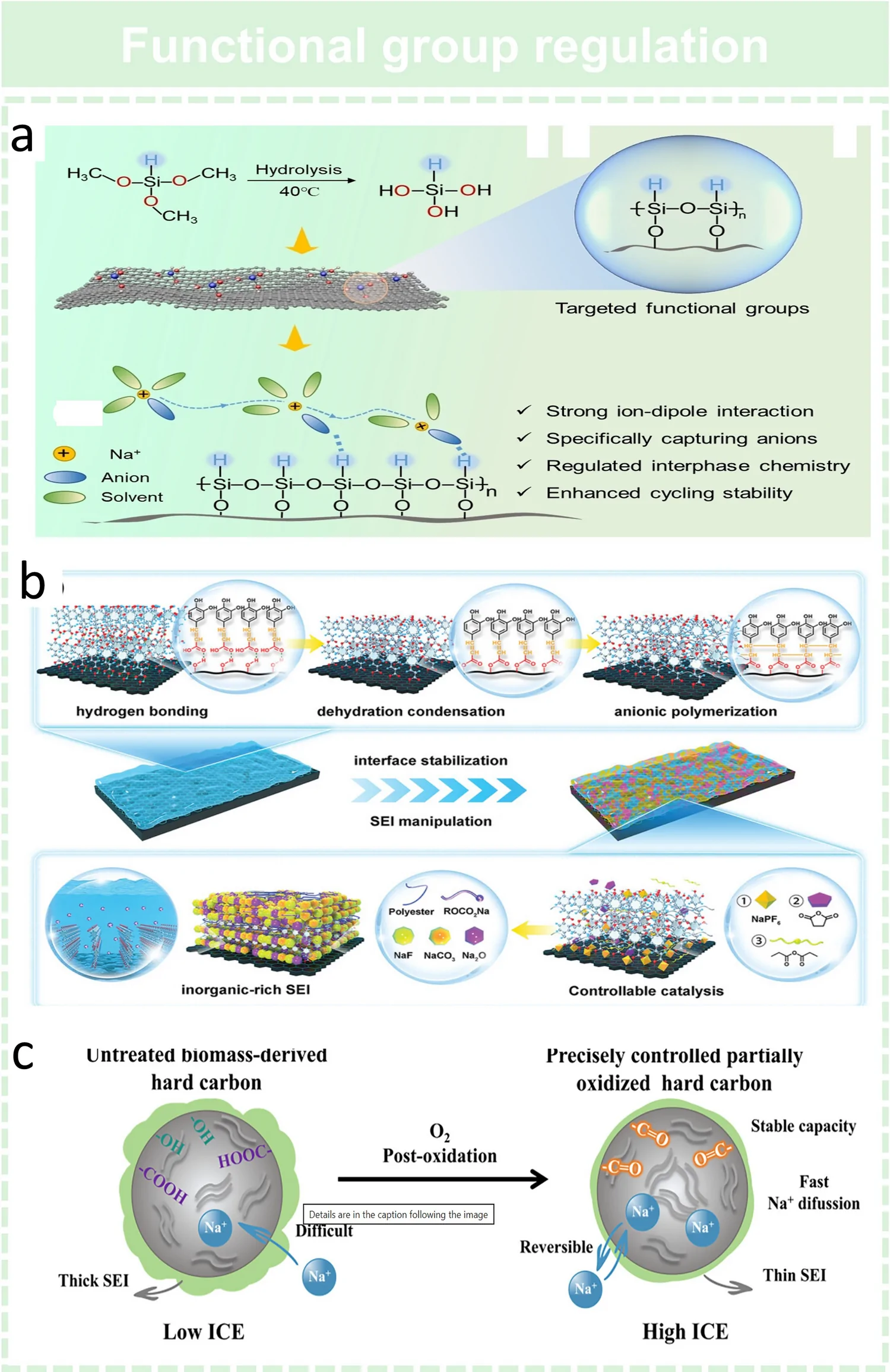
**a** Construction of the Si−O−Si molecular layer [167]. Copyright 2025, Wiley. **b** In situ grafting polymerized caffeic acid [169]. Copyright 2023, Wiley. **c** Partial oxidation strategy [170]. Copyright 2025, Wiley

As a representative example, Zhang et al. have exploited chemical bonding between trimethoxysilane (TMS) molecules and oxygen-containing functional groups on HC surfaces, ingeniously bridging to form a Si–O–Si molecular layer (TMS HC) and successfully introducing thiol functional groups (Fig. 15a) [167]. The inherently highly reactive oxygen-containing functional groups on HC are subtly converted into oriented functional groups capable of specifically anchoring anions. The enhanced ion dipole interactions between these oriented groups and PF_6_^−^ enrich anions at the HC electrolyte interface, giving rise to the ordered formation of a hybrid inorganic/organic SEI film. Experimental results demonstrate that after this treatment, the local molecular layer and electrolyte components collectively participate in SEI formation, leading to the generation of Na_2_SiO_3_. The content of NaF in the SEI film was markedly increased, which not only favors the formation of a thinner SEI film but also improves ionic conductivity owing to the reduced content of CO_3_^2-^. Similarly, Romero Cano et al. have employed melamine, urea, and citric acid to treat pomelo peel derived carbon at low temperatures, incorporating oxygen containing functional groups and nitrogen species [168]. This surface modification improves the electrolyte wettability on the material surface, suppresses the excessive formation of SEI films, promotes the SEI uniformity, and enhances the interfacial contact between HC and electrolytes while facilitating sodium ion insertion. Liu et al. have subjected mixed powders of HC and caffeic acid to dehydration condensation treatment at elevated temperatures by an ultrasonic mixed acid oxidation method, resulting in the formation of stable chemical bonds (e.g., carbonyl and carboxyl groups (Fig. 15b)) [169]. FTIR confirms the successful construction of carboxyl (–COOH) and carbonyl (C=O) functional sites in the carbon skeleton. These groups provide additional active sites and defects, enhance electron mobility, and endow the material with excellent rate capability. The optimized HC-CA 15% sample retains 82.1% of its capacity after 10,000 cycles at 5 A g^−1^, corresponding to an ultra-low-capacity fading rate of 0.0018%. Yang et al. have proposed a mild yet efficient partial oxidation strategy [170]. In practice, they subject the pristine HC material CS-H to heat treatment in air for 3h, precisely tailoring the surface chemistry of HC to achieve a carbonyl-dominated surface environment (Fig. 15c). The oxidized sample CS-HO delivers remarkably enhanced electrochemical performance. The ICE surges from 41.7% for the pristine counterpart to 70.3%.

Nonetheless, an excessive density of surface functional groups can trigger the proliferation of undesirable surface defects, impair the crystallinity and electrical conductivity of the material, and heighten the risk of side reactions between the electrode material and electrolyte, thus exerting a detrimental effect on the rate capability and cycling stability of SIBs. The design of surface functional group regulation strategies is fundamentally dictated by the intrinsic surface chemistry of the HC matrix, which is predetermined by upstream pretreatment and mid-pyrolysis processes, while also providing reverse design guidance for the full fabrication chain. The type and concentration of surface functional groups formed during upstream pretreatment and pyrolysis directly define the required functional group regulation strategy. Excessive oxygen-containing functional groups generated via pre-oxidation pretreatment or oxidative pyrolysis atmosphere require reductive surface functional group regulation to eliminate irreversible active sites, whereas HC fabricated via reductive atmosphere pyrolysis exhibits minimal surface functional groups, requiring oxidative functionalization to enhance electrolyte wettability. The pore architecture formed via upstream pore-forming pretreatment and pyrolysis also governs the effectiveness of surface functional group regulation. HC with a hierarchical interconnected pore framework enables uniform functional group modification across the entire material bulk, whereas dense HC with minimal open porosity only permits surface-only functionalization, with negligible impact on bulk properties. For HC with low ICE caused by excessive surface defects from uncontrolled pyrolysis, targeted surface functional group regulation can passivate irreversible active sites without altering the pre-optimized bulk pore and microcrystalline structure, achieving significant efficiency enhancement without compromising rate capability.

### Post-doping

Element doping refers to the introduction of foreign elements into HC materials via chemical or physical approaches to tailor their structure and performance [171]. Its primary objective is to modulate the electronic structure and chemical composition of the surface of HC, thereby enhancing its compatibility and wettability with electrolytes and further improving the adsorption and storage performance of sodium ions.

**Fig. 16:**
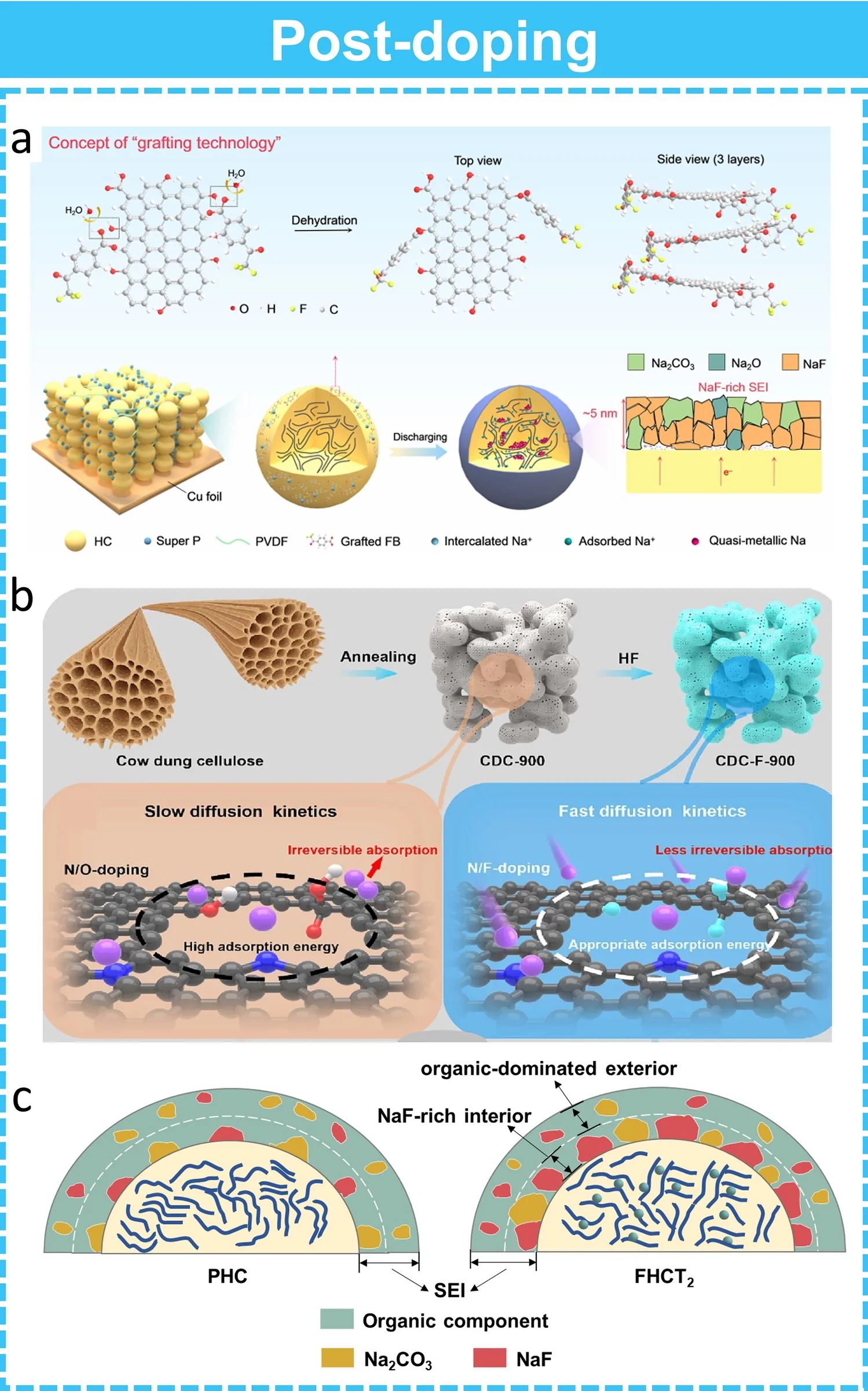
**a** Fluorine doping [174]. Copyright 2025, The Royal Society of Chemistry. **b** Influence of N/F co-doping on the structure and performance [175]. Copyright 2024, Elsevier. **c** Effect of F-doping on the physicochemical properties of HC [176]. Copyright 2024, Wiley

Surface fluorine doping is a commonly used doping strategy [172–175]. As illustrated in Fig. 16a, Sun et al. have regulated the electrochemical interfacial behavior of HC anodes by introducing the highly fluorinated molecule 4-(2,2,2-trifluoroacetyl) benzoic acid (FB) onto the surface of commercial HC, thereby significantly enhancing its sodium storage performance and cycling stability [174]. The researchers first anchor FB molecules onto the HC surface by dehydration condensation. The two C=O bonds in FB molecules can reversibly adsorb sodium ions through internal structural transformation to enhance the sodium storage capacity of HC in the sloping region. Meanwhile, FB molecules provide additional fluorine atoms to assist in constructing a stable NaF-rich SEI layer with a thickness of ~ 5 nm and minimizing the continuous consumption of electrolytes. The optimized fluorinated HC (FHC) achieves an ICE of up to 90.0% and a reversible sodium storage capacity of 359 mAh g^−1^ and can stably cycle for over 5000 times at a high current density of 2.0 A g^−1^. Beyond single-element doping, He et al. have reported N/F doped microporous carbon nanospheres prepared by simple annealing and HF solvothermal reaction, showing excellent rate capability and long-term cycling stability in sodium/potassium ion batteries (Fig. 16b) [175]. Yang et al. have mixed tetrafluoroterephthalic acid as the doping source with commercial HC to prepare fluorine-doped HC (Fig. 16c) [176]. They found that excessive F doping exacerbates the expansion of C-F bond lengths after sodiation, leading to structural distortion and accelerated capacity fading. The optimized sample with appropriate F doping exhibits a high capacity (434.53 mAh g^−1^) at 20 mA g^−1^ and excellent rate capability.

The design of post-doping strategies is fundamentally governed by the bulk and surface structure of the HC matrix, which is predetermined by upstream pretreatment and mid-pyrolysis processes, while also forming synergistic coupling with pre-doping implemented at the precursor stage. The pore architecture and defect density formed via upstream pretreatment and pyrolysis directly dictate the dopant type, doping method, and achievable doping level of post-doping. The defect density generated via pyrolysis also governs the effectiveness of post-doping, with intrinsic defect sites acting as nucleation centers for heteroatom incorporation, enabling stable doping without disrupting the carbon framework. Meanwhile, post-doping can be synergistically integrated with upstream pre-doping to achieve a bulk-surface gradient doping architecture, which is inaccessible via single-stage doping alone. Upstream pre-doping modulates the bulk electronic structure and interlayer spacing of the carbon matrix, while post-doping optimizes the surface interfacial properties, achieving concurrent enhancement of bulk ion diffusion kinetics and surface SEI stability. This synergistic doping strategy eliminates the performance tradeoffs associated with single-stage bulk doping, which often enhances rate capability at the expense of ICE. Furthermore, post-doping can compensate for the intrinsic limitations of upstream pretreatment and pyrolysis processes, without altering the pre-optimized bulk microstructure. For HC with poor rate performance caused by insufficient bulk conductivity from low-temperature pyrolysis, post-doping with electron-rich heteroatoms can enhance electronic conductivity without requiring re-optimization of the pyrolysis protocol, which would otherwise compromise the pre-optimized pore structure. In turn, the performance limitations of post-doping impose reverse design constraints on upstream pretreatment and pyrolysis processes. To achieve uniform bulk post-doping, upstream pretreatment and pyrolysis must be tailored to preserve interconnected mesoporosity, avoiding excessive pore closure during high-temperature carbonization.

### Pore Filling

The pore filling strategy centers on the directional sealing of excessive open pore channels and surface through holes via physical or chemical approaches [177]. The essence of this strategy is the reconstruction of pore connectivity and open-closed state, rather than fine tuning of the pore size dimension. The core objective of this strategy is to strictly constrain excessive open porosity, reduce the specific surface area of the material, suppress irreversible parasitic reactions between the carbon matrix and electrolyte, and convert partial open pore channels into closed pore cavities to amplify the capacity contribution from the low voltage plateau region.

**Fig. 17:**
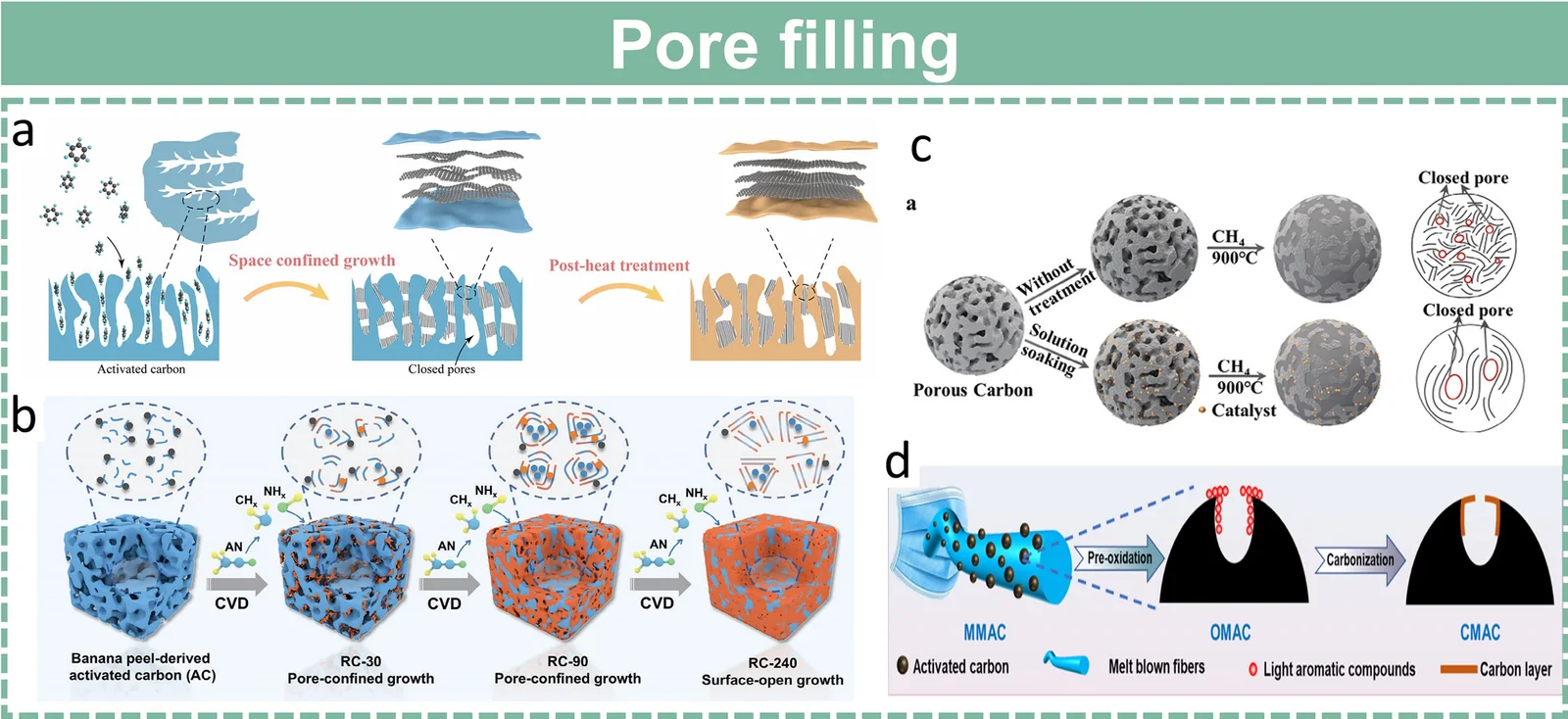
**a** Spatial confinement benzene filling [179]. Copyright 2023, The Royal Society of Chemistry. **b** Pore clogging induced by CVD [180]. Copyright 2024, Wiley. **c** CH_4_-assisted CVD [181]. Copyright 2025, Wiley. **d** Polypropylene CVD deposition [182]. Copyright 2024, Elsevier

Benzene, acetonitrile, methane, and other easily decomposable organic carbon sources are used as pore fillers [178]. Chen et al. have proposed a spatial confinement chemical vapor deposition strategy, where graphitoid carbon layers derived from benzene vapor are filled into the slit micropores of activated carbon, while simultaneously constructing graphitoid domains and microporous structures within the HC to yield filled carbon FC (Fig. 17a) [179]. The optimized FC-3h-1300 electrode delivers a high reversible capacity of 435.5 mAh g^−1^ at 20 mA g^−1^ and an exceptional cycling lifespan exceeding 1000 sodium storage cycles, albeit with inferior rate capability. In a separate study, Peng et al. engineered activated carbon derived from biomass waste banana peels via an acetonitrile-mediated pore-filling strategy. Using acetonitrile as the carbon and nitrogen source, they in situ deposited graphitic carbon and stacked N-doped pseudo-graphitic layers at the mouths of open pores, enabling the in situ conversion of open pores to closed pores. This structural modification delivers a remarkable enhancement in the sodium storage capacity of sodium-ion batteries (Fig. 17b) [180]. Their findings indicate that insufficient deposition duration hinders the transformation of open pores to closed pores suitable for sodium storage, whereas excessive deposition time leads to complete pore blockage and over-graphitization, thereby impeding sodium ion transport channels. Notably, Deng et al. have utilized methane CH_4_ as the gaseous precursor and porous carbon as the substrate, with Fe catalysts employed to lower the CH_4_ decomposition energy barrier (Fig. 17c) [181]. This approach improves the pyrolytic carbon deposition efficiency (deposition time shortened by 25%) while promoting carbon atom rearrangement to form expanded graphitic microdomain structures. Zhang et al. have found that waste masks polypropylene PP undergo pyrolysis to generate small molecule aromatic compounds under the catalysis of activated carbon (Fig. 17d) [182]. Meanwhile, the small molecule aromatic compounds produced by PP pyrolysis are deposited and coated on the surface of AC, converting the open pores of commercial AC to closed pores. The optimized CMAC electrode achieves an ICE of 88.7%. Li et al. also have converted waste lunch boxes into small aromatic compounds to seal the open pores of biomass-derived porous carbon [183].

Pore-filling not only facilitates surface coating but also allows for the accurate repair and reconstruction of pore architectures, such as transforming open pores into closed configurations. Its principal advantages reside in exceptional conformality and tunability; gaseous precursors can infiltrate deeply within porous carbon matrices, enabling uniform modification throughout the substrate. Nevertheless, the pore-filling process is notably intricate. Subtle variations in temperature, pressure, precursor flow rate, and duration profoundly influence the morphology of the deposited carbon, which may range from amorphous to graphene-like, as well as its precise location at the pore mouth or along the inner pore wall. Excessive deposition can obstruct ion transport pathways, whereas insufficient deposition may lead to inadequate functional outcomes. Consequently, the successful implementation of pore-filling hinges critically on a comprehensive understanding of the initial pore structure of the carbon substrate coupled with meticulous control over deposition kinetics. The pore structure characteristics predetermined by pre-treatment and mid-process carbonization exert a deterministic influence on pore filling process design, precursor selection, and parameter optimization. The pore size distribution, open porosity, and pore interconnectivity formed via upstream pore-forming pretreatment directly dictate the type of gaseous precursor and deposition kinetics required for effective pore occlusion. Excessive microporosity generated via uncontrolled activation pretreatment will inevitably lead to complete pore blockage during CVD deposition, which severely compromises the rate capability of the final material.

### Surface Coatings

The high specific surface area of HC induces excessive decomposition of the electrolyte on its surface during the initial charging-discharging process, leading to the formation of unstable solid electrolyte interphase SEI films and irreversible capacity loss. Consequently, the ICE is generally below 75% [184].

**Fig. 18:**
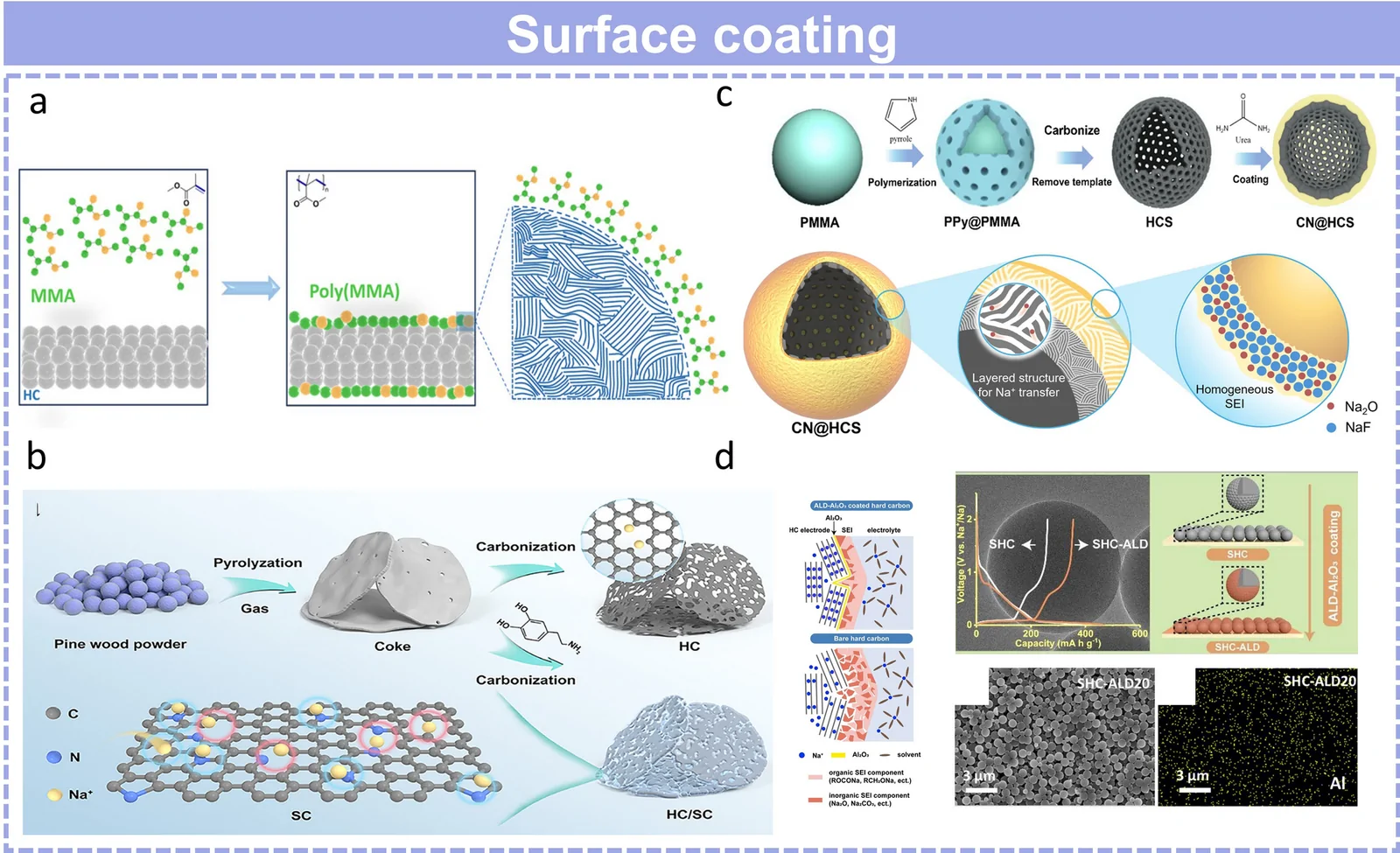
**a** Schematic illustration of MMA polymerization on HC [186]. Copyright 2025, Wiley. **b** PDA-derived soft carbon coating [187]. Copyright 2025, Wiley. **c** g-C_3_N_4_ coating [188]. Copyright 2025, Wiley. **d** ALD-Al_2_O_3_ atomic layer [189]. Copyright 2019, Elsevier

Surface coating techniques, encompassing approaches such as atomic layer deposition ALD and soft HC composites, are used to deposit continuous and dense protective layers, such as carbon layers, metal oxides, or conductive polymers, on the HC surface. This effectively impedes electrolyte penetration, enhances interfacial mechanical stability, and improves electrochemical characteristics to boost the overall battery performance [185]. For instance, Zhang et al. have fabricated a poly-methyl methacrylate Poly-MMA-based pure organic SEI layer on the surface of commercial Type 1 HC (Fig. 18a) [186]. This interfacial structure reconfigures the Na^+^ DME solvation shell into a Na^+^ DME Poly-MMA microenvironment, and such interfacial reconstruction significantly facilitates desolvation of sodium ions and interfacial transport kinetics. In another study, Cui et al. have modified the HC surface with a soft carbon coating derived from polydopamine PDA, which covers the open pores on the HC surface and reduces the direct contact between the electrolyte and the HC surface (Fig. 18b) [187]. Furthermore, Jiao et al. have thermally deposited an electronically inert graphitic carbon nitride g-C_3_N_4_ layer on hollow carbon spheres CN@HCS, which effectively reduces the excess specific surface area and shields structural defects, thereby suppressing undesirable electrode-electrolyte side reactions (Fig. 18c) [188]. The g-C_3_N_4_ on CN@HCS efficiently promotes the absorption and reduction of fluoroethylene carbonate FEC, forming a uniform, robust, and inorganic-rich SEI layer. The abundant π conjugated electronic system and negative charge centers in g-C_3_N_4_ provide sufficient and rapid migration channels for charge transport. Cao et al. have employed phenolic resin as the raw material and deposited an ultra-thin aluminum oxide Al_2_O_3_ film on the HC electrode surface by atomic layer deposition (ALD) (Fig. 18d) [189]. This film serves as an artificial solid electrolyte interphase SEI, which inhibits electrolyte decomposition to improve the ICE and cycling stability. Meanwhile, the ultra-thin Al_2_O_3_ film deposited on the reduces the interfacial resistance and electrode overpotential. At 20 mA g^−1^, the reversible specific capacity and ICE reach 355.5 mAh g^−1^ and 75.5%, respectively. Nevertheless, the high cost of ALD renders it unamenable to large-scale industrialization. Subsequently, researchers have developed a simple liquid phase coating method to form a homojunction Al_2_O_3_ coating layer [190]. This approach effectively reduces the active sites, such as defects, pores, and functional groups, on the HC surface, thereby suppressing the occurrence of side reactions and continuous electrolyte decomposition, and forming a thinner SEI film with fewer inorganic components.

Despite the versatility of surface coating with various materials, it faces inherent limitations [191, 192]. For carbon coating layers, the bonding with the HC matrix may be insufficient, leading to easy detachment during subsequent electrode processing or battery cycling and consequent performance degradation. For metal oxide coating layers, while they can enhance the chemical stability of materials and reduce side reactions with electrolytes, they may increase material weight and lower battery energy density. For polymer coating layers, they possess good flexibility and adhesion, enabling uniform coverage on HC particle surfaces, but their relatively poor electrical conductivity requires composite use with other conductive materials to achieve optimal performance. Additionally, the chemical stability of the surface coating layer must be compatible with the electrolyte, and precise control of coating thickness and uniformity remains challenging. An excessively thick coating layer may impede sodium ion diffusion and compromise battery rate capability.

### Pre-sodiation

Pre-sodiation refers to the process of pre-introducing sodium ions into the HC anode by various approaches prior to battery assembly [193]. Its core principle lies in compensating for sodium ions lost by HC during the initial cycle due to reactions with the electrolyte, thereby substantially enhancing the ICE and reversible capacity of SIBs [194]. Fundamentally, pre-sodiation occupies partial sodium storage sites within HC materials. This enables more sodium ions to effectively participate in electrochemical reactions during subsequent actual charge discharge cycles, thus optimizing the overall performance of the battery [195].

#### Physical Pre-Sodiation

Physical pre-sodiation generally entails directly adhering sodium powder or sodium foil to the electrode surface via mechanical roll pressing under a specific pressure, or incorporating sodium powder into the electrode slurry for electrode fabrication, thereby enabling the straightforward realization of pre-sodiation. While it features simple operation, it imposes stringent requirements on the environment and poses considerable challenges to processing technology. Additionally, excess sodium metal on the surface can increase the polarization degree of the battery.

**Fig. 19:**
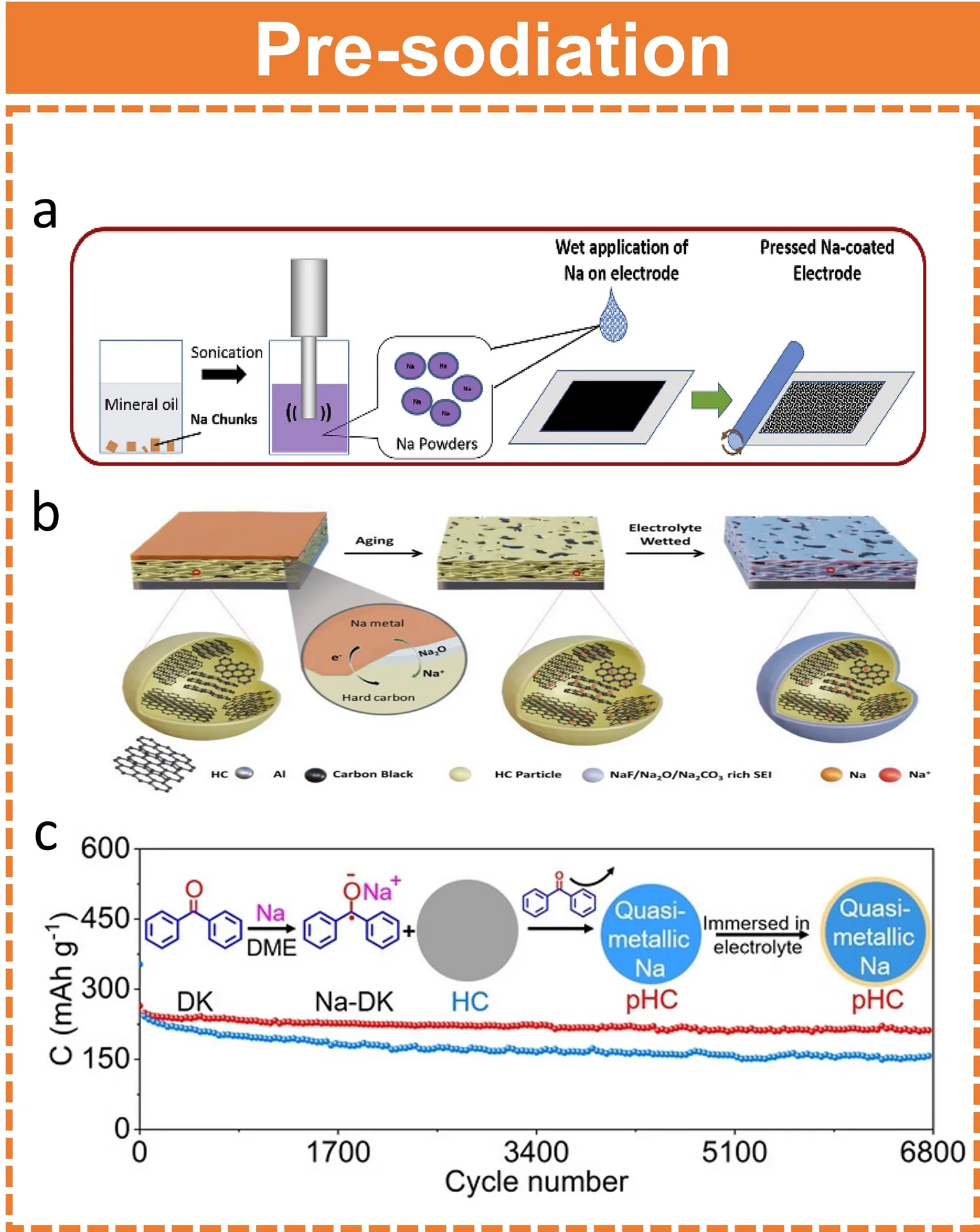
**a** Physical pre-sodiation strategy [197]. Copyright 2018, Elsevier. **b** Schematic diagram of uniform deposition pre-sodiation for HC electrode [198]. Copyright 2024, Elsevier. **c** Na-DK chemical pre-sodiation [200]. Copyright 2022, Wiley

For instance, Liu et al. achieved pre-sodiation by direct roller pressing contact between sodium metal sheets and anode sheets [196]. Through compensating for the irreversible loss of active materials via sodium metal during cycling, the ICE increases significantly from 24% to 75% and remains at 99% in subsequent cycles. However, metallic sodium is highly reactive and difficult to keep stable in air. Drawing inspiration from the stable lithium metal powder used in pre-lithiation, Tang et al. have dispersed molten metallic sodium in mineral oil using pulsed ultrasound in an inert atmosphere (Fig. 19a) [197]. The cleaned and stabilized sodium powder is suspended in n-hexane and adheres to the carbon anode surface by roller pressing after drying. This pre-sodiation approach reduces the irreversible capacity ratio from 19.3% to 8% and also achieves a 10% capacity increase and 5% energy density improvement in full cells. In another innovative approach, Wang et al. have fabricated uniformly pre-sodiated HC by solid-state electrochemical reactions between HC and pre-deposited metallic sodium films without introducing liquid electrolytes (Fig. 19b) [198]. They deposit metallic sodium films of desired thickness on the HC anode surface via vacuum thermal evaporation, enabling precise control of the pre-sodiation degree and avoiding the hazards of metallic sodium. Upon immersion in electrolyte, an artificial SEI film rich in inorganic components is formed on the surface of pre-sodiated HC due to spontaneous chemical reactions, increasing the ICE of HC from 76.0% to 107.9%. The Na_2_O formed between sodium and HC is a product of the chemical reaction between sodium and adsorbed O_2_ on HC, which provides ion channels for Na⁺ transport.

#### Chemical Pre-sodiation

Chemical pre-sodiation refers to a method involving chemical sodium supplementation of electrode materials for sodium ion batteries using strongly reductive sodiation reagents. This approach enables precise regulation of the pre-sodiation depth by controlling reaction temperature, reaction time, as well as the type and concentration of sodiation reagents. However, it is difficult to avoid the impact of residual reagents on performance [32]. Meng et al. have leveraged the thermal decomposition of NaBH_4_ to generate metallic sodium on the HC surface for sodium loss compensation, fabricating HCP. Sodium occupies intrinsic irreversible sites, thereby reducing irreversible capacity [199]. Fang et al. have prepared bifunctional pre-sodiation of HC anodes using sodium benzophenone Na-DK [200]. It compensates for irreversible sodium adsorption on oxygen-containing functional groups and reacts with five-membered/seven-membered ring carbon defects in HC to form quasi-metallic sodium. In 1.0 M NaPF_6_ in G2 electrolyte, the formed sodium induces the formation of a stable NaF-rich SEI on HC, which is beneficial for interfacial reaction kinetics and stable Na^+^ intercalation and deintercalation (Fig. 19c). This endows the pre-sodiated HC with a high ICE of ~ 100% and a capacity retention rate of up to 82.4% after 6,800 cycles. Full cells paired with Na_3_V_2_(PO_4_)_3_ cathodes exhibit a high-capacity retention rate of 100% after 700 cycles. Nevertheless, research on electrochemical pre-sodiation of HC remains relatively scarce and warrants further in-depth exploration.

Despite the enormous potential of pre-sodiation in enhancing the performance of HC anodes, it still faces several challenges in practical applications. First and foremost, safety issues during the pre-sodiation process constitute a critical issue requiring urgent resolution. Metallic sodium possesses extremely high chemical reactivity and readily undergoes vigorous reactions with moisture and oxygen in the air, potentially leading to hazards such as combustion and explosion [201]. Second, precise control of the pre-sodiation degree remains a major challenge. Different pre-sodiation methods and process parameters result in variations in the distribution and content of sodium ions in HC materials, and either excessive or insufficient pre-sodiation may exert adverse effects on battery performance. Thus, it is necessary to establish precise methods and technical standards for pre-sodiation degree control to optimize and stabilize pre-sodiation effects. Furthermore, the pre-sodiation process may introduce additional impurities or cause certain damage to the original structure of HC materials. These factors may also affect the long-term performance and stability of batteries, requiring in-depth investigation and resolution in subsequent research.

This section systematically evaluates the regulatory effects of diverse post-treatment strategies on the interfacial properties and electrochemical performance of HC anodes. The implementation of post-treatment protocols is not a standalone remedial step for performance optimization, but exhibits tight bidirectional coupling with both upstream pretreatment and carbonization stages. The effectiveness of post-treatment is fundamentally constrained by the intrinsic microstructural features formed in the upstream pretreatment and carbonization stages. Conformal coating via CVD requires a moderate uniform specific surface area generated via coordinated pretreatment and carbonization. For post-treatment targeting high ICE via surface functional group regulation, upstream pretreatment must strictly control the formation of irreversible oxygen containing functional groups. Carbonization protocols must also limit the generation of open porosity and surface dangling bonds in this scenario. Isolated post-treatment modification can only deliver marginal performance improvements while often introducing additional kinetic barriers for ion transport. A common example is excessive surface coating without addressing the intrinsic structural defects generated in upstream stages. This demonstrates that post-treatment must be integrated into the holistic full chain design framework from the initial stage of process development, rather than applied as an after the fact remedial measure.

## Quality Control of Commercial HC

### Full Chain Collaborative Preparation of Commercial HC

Currently, there are indeed many new energy technology companies selling HC, such as BTR, Shanshan Group, ShengQuan Group, Kuraray, Sichuan BSG, Carmery Wuhu, JNION Energy, RongNa New Energy, NK new materials, etc. Unlike the isolated regulation process in the laboratory, commercial HC primarily focuses on the stability of the shipped products, rather than solely on improving the performance of a single indicator. While each company has its own unique technological approach, the commonality is that all processes must be coordinated throughout the entire process to obtain high-quality HC products.

Kuraray is the earliest representative HC product. In the research and development process of HC, many companies use Kuraray’s HC products as benchmark samples. Currently, many domestic HCs have surpassed Kuraray in terms of production capacity and quality. Kuraray’s HC uses natural coconut shells from Southeast Asia as raw materials, which requires high consistency of raw materials. After multiple processes such as raw material screening, pre-carbonization, crushing, alkali impregnation, purification, and CVD treatment, a HC product with low impurities and consistent and stable performance is obtained. Similarly, using biomass as raw material, ShengQuan Group adopted a different technological route and chose straw as the raw material, which is lower in cost. ShengQuan leveraged its advantages in biomass refining and resin synthesis technology to invent the ShengQuan Biomass Solvent Method, which comprehensively utilizes straw biomass to transform it into bio-based resin, and further adopts a two-step carbonization process to form HC. They first selectively dissolve lignin, some hemicellulose, and cellulose, which have high carbon content and are easy to form carbon, into a biological solvent, and then control these substances to undergo intermolecular and intramolecular rearrangement in the solvent, allowing the migrated molecules to completely dissociate and detach from the original system. These substances rearrange and connect to form bio-based resin, then pre-carbonize to form biomass carbon, and finally calcine and carbonize to form HC. NK new materials company utilizes biomass as the raw material. Initially, the molecular weight and crystallinity of the raw material are controlled. Subsequently, through techniques such as precursor cross-linking and solidification, pre-carbonization, morphology regulation, surface pre-oxidation modification, surface coating, and purification, the sodium storage performance of biomass HC anodes is effectively enhanced. Biomass raw materials are commonly used in commercial HC, but their consistency is poor.

Carmery is the only enterprise in China that focuses on the “coal-based HC” technology route and achieves kiloton-scale production, utilizing the technology from Chengmeng Chen’s team at the Chinese Academy of Sciences. They have a deep partnership with Middling Coal Huali, using high-carbon, low-ash coal as raw material, with abundant petrochemical feedstock. The core essence of the technology is to overcome the tendency of coal-based raw materials to graphitize easily. At the same time, they have developed a spherical preparation strategy for HC microspheres to enhance compaction density.

BSG New Energy Co., Ltd., adopts a different approach from previous companies, utilizing a multi-precursor technology route and a gradient carbon coating process. Currently, they offer a wide range of product models, covering both high-capacity and high-power HCs. They do not rely on a single raw material, but flexibly select from biomass coconut shells, starch, phenolic and urea–formaldehyde resins, and mixtures of plastic, biomass, and coal materials as raw materials based on specific performance indicators and cost requirements. Additionally, they employ a gradient composite carbon coating process, initially filling the micropores of porous carbon with small molecular carbon sources (such as methane) through CVD, and then coating the outer layer with large molecular carbon sources. This process not only effectively seals the pores (improving initial efficiency) but also results in a smoother particle surface, enhancing compaction density.

### Consistency Control of Commercial HC

The core goals of laboratory research and industrial production of HC are different [1]. The laboratory pursues ultimate performance, with the core being to solve the precise structure–activity relationship between structure and performance, while industry pursues stable and low-cost mass production, facing triple challenges of materials, processes, and equipment. In the laboratory, extreme parameter exploration (such as ultra-fast heating rate and extremely short reaction time) can be achieved to study the impact of a single variable on performance, while in engineering, the process parameter window is narrower, achieving a balance between controllability and stability, relying on advanced equipment to achieve precise zone temperature control and dynamic sealing [30]. The laboratory is the starting point for structural innovation in HC materials, and industrial production is the path to realizing the value of HC. To achieve product consistency in engineering, it is necessary to ensure the consistency of raw materials and ensure that equipment can operate continuously and stably under certain process conditions.

#### Raw Material Control

In terms of materials, it is necessary to maintain stable supply of raw materials and consistency of raw material components. Engineering requires compromise with raw materials to a certain extent and cannot pursue the use of high-purity reagents and raw materials like in the laboratory. In engineering, the first step is to strictly control the source of raw materials, such as locking in biomass specific origin, variety, and maturity of raw materials, and even requiring suppliers to cultivate specialized varieties as needed [30]. At the same time, it is necessary to conduct on-site testing of physical and chemical indicators such as moisture, ash content, and volatility for each batch of raw materials to control the range of these physical and chemical indicators. For natural raw materials, artificial intervention through physical and chemical means is used to reshape consistency. Clean and remove impurities from the raw materials by rinsing and soaking to remove surface soil, and after crushing, remove impurities through equipment such as vibrating screens and magnetic separators. Crush the raw materials uniformly to the target particle size to ensure uniform reaction activity. At the micro level, the differences in raw materials can be smoothed out, for example, by mixing and reacting acid solution, crosslinking agent, and raw materials to make their physical and chemical properties similar, laying a uniform foundation for subsequent uniform carbonization [202–208].

#### Device Control

In terms of equipment, in addition to crushing and screening equipment, heating and CVD coating equipment are the core, and the equipment needs to serve the process. Unlike laboratory experiments that use small tube furnaces or Joule heating equipment to heat raw materials, engineering production requires equipment with automation, continuous, dynamic sealing, uniform thermal field, and intelligent compensation capabilities to ensure consistency of HC products. These devices need to meet uniform mass and heat transfer requirements under large-scale material conditions. In the HC engineering, continuous production equipment is needed to replace traditional intermittent equipment, and rotary kilns and roller furnaces are two common types of equipment [203]. The core advantage of continuous rotary kiln lies in the continuous rolling of materials in the furnace, uniform heating, which is very suitable for carbonization, activation, and other processes that require dynamic mixing and efficient heat transfer. Through variable frequency speed regulation, the residence time of materials can be accurately controlled to meet different process requirements. The advantage of continuous roller furnace lies in smooth conveying, especially suitable for static heat treatment such as coating and pyrolysis of powder or granular materials [30]. Through independent temperature control in multiple temperature zones, excellent temperature uniformity can be achieved to ensure product consistency. Its modular design also facilitates flexible adjustment of production capacity, which is more conducive to the production of homogeneous HC with a capacity of 10,000 tons. The continuous kiln overcomes the problem of difficult repetition of process conditions and poor product consistency caused by circulation gaps such as loading, heating, and cooling in traditional batch production. At the same time, a high-precision dynamic sealing system is adopted, combined with intelligent pressure compensation technology, to eliminate material oxidation and pollution risks from the source, control temperature uniformity within a very small range, and adopt a multi zone independent temperature control system to ensure that materials react under the optimal temperature curve.

## Conclusions and Prospects

**Fig. 20:**
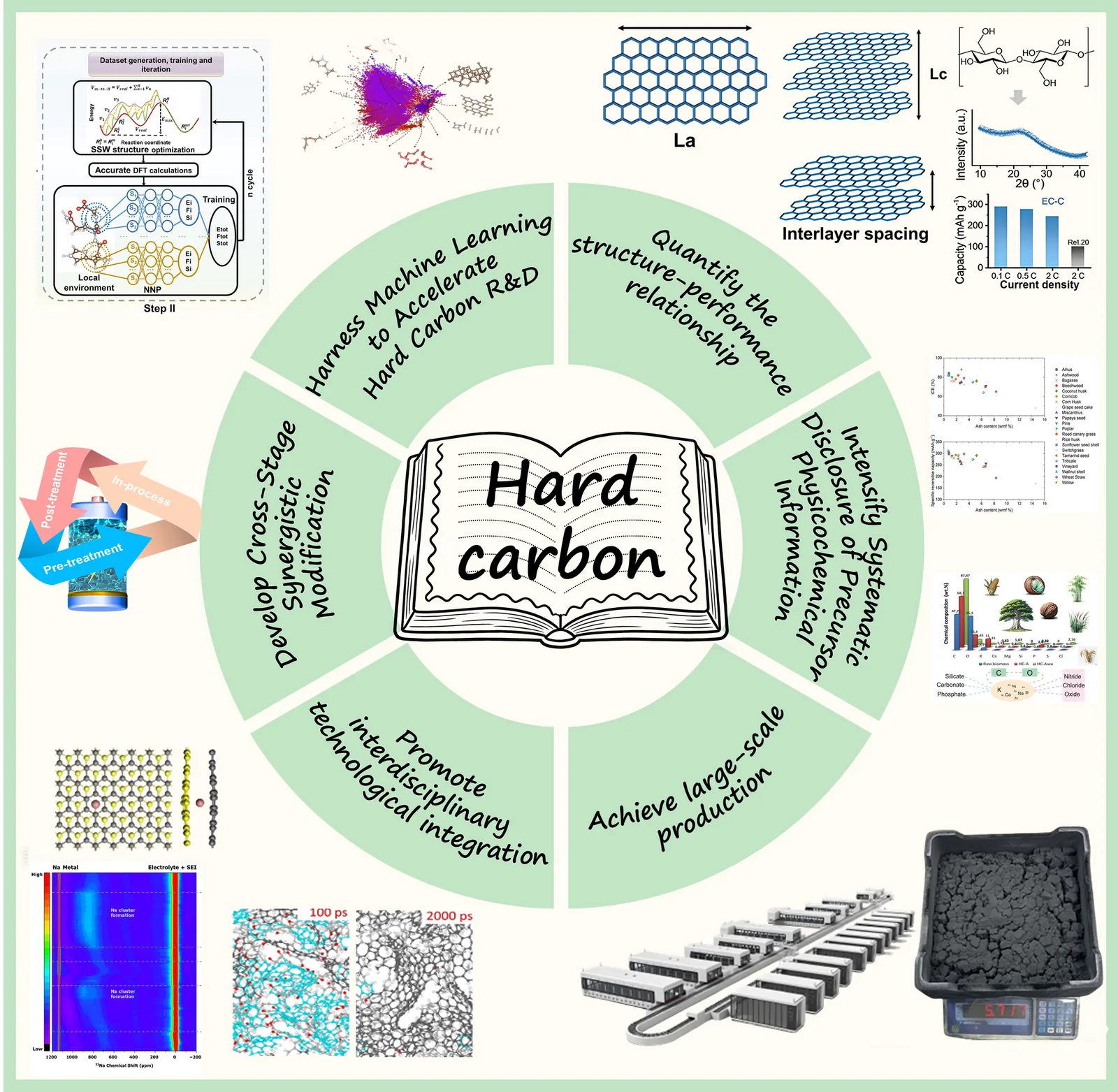
Future development perspectives of HC [1, Copyright 2025, Wiley. 33, Copyright. 2025, Elsevier. 104, Copyright 2025, The Royal Society of Chemistry. 197, Copyright 2026, Elsevier.^198^, Copyright 2024, Wiley]

The diversity of HC precursors and the versatility of fabrication and optimization methodologies endow HC with exceptional microstructural tunability, while simultaneously exacerbating the inherent intricacies of targeted modification. Compounded by the fragmented research paradigm centered on single-point optimization, this scenario has long impeded the rational design of high-performance HC anodes, where isolated advances in precursor screening, pyrolysis regulation, or post-treatment implementation frequently yield suboptimal outcomes owing to neglected synergistic interactions across the full fabrication chain. Against this backdrop, the present review first elucidates the collective regulatory mechanisms through which four core structural attributes of HC, namely graphitic nanodomains, structural defects, morphological features, and nanoporous architectures, govern the distinctive dual sodium storage behaviors corresponding to slope and plateau regions. Subsequently, this work systematically dissects the technical pathways and theoretical underpinnings of HC modification from the holistic perspective of full process engineering, encompassing pretreatment protocols such as hydrothermal processing and pre-oxidation, mid-process control strategies including conventional pyrolysis and flash Joule heating, and post-treatment tactics like surface coating and pre-sodiation. Furthermore, it critically appraises and comparatively evaluates the respective merits, limitations, and interdependencies of these strategies, clarifying how treatment effects at one fabrication stage precondition the performance outcomes of subsequent stages. Nevertheless, the full scope of synergistic mechanisms spanning the entire fabrication chain remains not fully elucidated. To promote the long-term development of SIBs energy storage applications, several new and effective viewpoints are proposed (Fig. 20).

### Establishing Multi-scale Quantitative Structure–Performance Relationships

Employing a suite of multi-dimensional characterization techniques including BET, FTIR, XRD, TEM, XPS, Raman, and NMR enables the establishment of a quantitative mapping model integrating pretreatment parameters such as pre-carbonization temperature, cross-linker molar ratio and pore content, dynamic carbonization conditions, HC microstructural parameters covering graphitic crystallite *Lc* and La, pore size distribution and defect-type proportion, and core sodium storage performance metrics, including reversible capacity, ICE, rate capability and cycling stability. This model provides a robust theoretical footing for the rational structural design of HC anodes and focuses on clarifying intrinsic correlations between structural parameters across atomic, mesoscopic, and macroscopic scales and sodium storage metrics, rather than relying on superficial qualitative descriptions. Specifically, at the atomic scale, carbon layer stacking modes, interlayer spacing, and defect types directly determine sodium ion intercalation sites and migration energy barriers, thus regulating the reversible capacity and rate performance of HC. At the mesoscopic scale, pore structure parameters, including pore size distribution, pore volume, and interconnectivity, regulate sodium ion accommodation space and diffusion pathways, which correlate closely with ICE and long-term cycling stability. At the macroscopic scale, particle morphology, tap density, and conductive network structure modulate the ion and electron transport kinetics of electrode materials, further regulating the overall electrochemical performance.

### Developing Cross-stage Synergistic Modification Strategy

Breakthrough of the performance limitations of single stage modification via systematic elucidation of interfacial coupling effects and synergistic interplay among pre-treatment, mid-process control and post-treatment is necessary. An integrated modification strategy featuring precursor structure customization, dynamic carbonization regulation and precise interface modification should be established to achieve synergistic optimization of multiple core performance objectives, including interlayer spacing modulation to facilitate sodium ion intercalation, pore structure synergy for balanced ion accommodation and transport, defect engineering to tailor redox active sites, and interface stabilization to suppress parasitic side reactions.

This integrated approach capitalizes on the complementary merits of technologies across disparate preparation stages by bridging the intrinsic disconnect between individual modification steps, ensuring that the regulatory effects of each stage reinforce rather than offset one another. Such a full process synergistic strategy facilitates the achievement of superior sodium storage performance in HC anodes, thereby propelling the advancement of high-performance materials tailored for practical SIBs.

### Promoting Interdisciplinary Technological Integration

Leveraging the synergistic strengths of computational simulation, advanced characterization, and materials science to advance interdisciplinary technological integration addresses critical mechanistic and performance bottlenecks in HC anode research. Specifically, integrating density-functional theory (DFT) calculations, molecular dynamics (MD) simulations, and in situ measurements unveils intrinsic mechanisms inaccessible to experimental approaches, including electronic structure evolution of HC, sodium ion adsorption and diffusion behavior at the atomic scale, and dynamic carbon skeleton rearrangement during pyrolysis. This integration enables seamless correlation between atomic-scale theoretical predictions and real-time experimental observations, validating simulation models while furnishing direct mechanistic evidence for the structure–property relationships of HC. Future research should establish interdisciplinary collaboration platforms to integrate theoretical modeling, computational simulation, in situ characterization, and performance validation, overcoming the limitations of isolated technology employment. Ultimately, this holistic approach will deepen fundamental insights into HC pyrolysis and sodium storage mechanisms, guide the rational design of high-performance HC anodes, and expedite the commercialization of sodium ion batteries in large-scale energy storage scenarios.

### Addressing Engineering Bottlenecks in Large-Scale Fabrication

Addressing engineering bottlenecks in large-scale fabrication stands as a pivotal prerequisite for translating high-performance HC anodes from laboratory-scale trials to practical energy storage applications. Current large-scale production of HC is plagued by unresolved challenges, including batch-to-batch performance inconsistency, high precursor and processing costs, poor compatibility with existing battery manufacturing lines, and insufficient scalability of laboratory-derived modification strategies. These bottlenecks arise from the mismatch between laboratory-derived small batch operations and industrial-scale continuous production demands, hampering the commercialization of HC-based SIBs. In line with practical application requirements, efforts should focus on developing low-cost, low-energy consumption, and environmentally benign large-scale modification technologies tailored for industrial scenarios. Examples include continuous liquid phase coating production processes, environmentally friendly protocols for mild pre-oxidation, and safe control technologies for low-cost pre-sodiation. Concurrently, attention should be paid to addressing equipment compatibility challenges of advanced carbonization technologies, which facilitate the translation of laboratory-scale performance into engineering applications by bridging the gap between academic process development and industrial operating parameters.

### Systematic Disclosure of Precursor Physicochemical Information

Intrinsic physicochemical properties of precursors stand as a core prerequisite for dictating pyrolysis pathways and final microstructural architectures of HC anodes, with key parameters including precise chemical composition, crystallinity, ash content, functional group species, and their spatial distribution exerting dominant effects on the evolution of carbon skeletons and pore structures during thermal conversion. Future research should establish standardized characterization criteria for the fundamental physicochemical parameters of precursors and fully disclose comprehensive critical information encompassing their raw material origins, specific pretreatment protocols, and detailed key physicochemical indexes such as elemental composition ratios, thermal stability thresholds, surface functional group densities, crystalline phase content, ash composition types, and spatial distribution of active sites. Such standardized characterization and full information disclosure lay a robust theoretical and experimental foundation for formulating tailored modification strategies targeting distinct precursor systems, while ensuring the reproducibility of research findings across different laboratories and experimental platforms, thereby effectively resolving the discrepancy in research conclusions caused by ambiguous, incomplete, or unstandardized disclosure of precursor information in current HC-focused studies.

### Harnessing Machine Learning to Accelerate HC R&D

Leveraging the inherent merits of machine learning in high-throughput screening process parameter prediction and structure-property relationship modeling, researchers are encouraged to integrate massive datasets covering precursor properties preparation processes and electrochemical performance metrics. This integration enables the establishment of intelligent prediction models that realize rapid optimization of preparation parameters for high-performance HC and efficient screening of novel precursors. Such an approach replaces the traditional R&D paradigm relying on empirical trial and error, thus drastically shortening R&D cycles and reducing associated costs.
